# Prognostic heterogeneity of Ki67 in non‐small cell lung cancer: A comprehensive reappraisal on immunohistochemistry and transcriptional data

**DOI:** 10.1111/jcmm.18521

**Published:** 2024-07-17

**Authors:** Yujing Yang, Xinye Shao, Zhi Li, Lingyun Zhang, Bowen Yang, Bo Jin, Xuejun Hu, Xiujuan Qu, Xiaofang Che, Yunpeng Liu

**Affiliations:** ^1^ Department of Medical Oncology The First Hospital of China Medical University Shenyang China; ^2^ Key Laboratory of Anticancer Drugs and Biotherapy of Liaoning Province The First Hospital of China Medical University Shenyang China; ^3^ Clinical Cancer Research Center of Shenyang The First Hospital of China Medical University Shenyang China; ^4^ Department of Oncology, Nanfang Hospital Southern Medical University Guangzhou China; ^5^ Department of Respiratory and Infectious Disease of Geriatrics The First Hospital of China Medical University Shenyang China

**Keywords:** Ki67, non‐small cell lung cancer, prognosis, tumour microenvironment, tumour proliferation

## Abstract

In the present study, the debatable prognostic value of Ki67 in patients with non‐small cell lung cancer (NSCLC) was attributed to the heterogeneity between lung adenocarcinoma (LUAD) and lung squamous carcinoma (LUSC). Based on meta‐analyses of 29 studies, a retrospective immunohistochemical cohort of 1479 patients from our center, eight transcriptional datasets and a single‐cell datasets with 40 patients, we found that high Ki67 expression suggests a poor outcome in LUAD, but conversely, low Ki67 expression indicates worse prognosis in LUSC. Furthermore, low proliferation in LUSC is associated with higher metastatic capacity, which is related to the stronger epithelial‐mesenchymal transition potential, immunosuppressive microenvironment and angiogenesis. Finally, nomogram model incorporating clinical risk factors and Ki67 expression outperformed the basic clinical model for the accurate prognostic prediction of LUSC. With the largest prognostic assessment of Ki67 from protein to mRNA level, our study highlights that Ki67 also has an important prognostic value in NSCLC, but separate evaluation of LUAD and LUSC is necessary to provide more valuable information for clinical decision‐making in NSCLC.

## 1 INTRODUCTION

Lung cancer has the highest morbidity and mortality worldwide,^1^ with the majority of patients being diagnosed with non‐small cell lung cancer (NSCLC). Despite great efforts in the exploration of new tumour biomarkers and development of treatment options, owing to the heterogeneity of NSCLC, most prognostic biomarkers have not been observed to show robust predictive performance in practice, leading to insufficient risk stratification of patients, and the survival burden of NSCLC remains large. Therefore, one of the critical unmet needs is to distinguish patients with different prognoses, for which an effective biomarker‐guided survival discrimination would significantly affect the treatment strategy and prognostic assessment for NSCLC in the era of precision medicine.

Sustaining uncontrolled proliferation is the most fundamental trait of malignant tumours,^2^ and the accumulating genomic instability would further lead to a growing tumour burden, organ infiltration and cachexia, adversely affecting the outcome. Ki67 is a protein encoded by the MKI67 gene, and its expression in the nucleus is cell‐cycle dependent and is involved in the whole phase from G1 to M.^3^, ^4^, ^5^ Notably, as the most classical biomarker used in assessing active tumour proliferation, Ki67 has been routinely applied for the immunohistochemical evaluation of malignancy and prognostic judgement in breast and neuroendocrine cancers. According to the recommendations of the International Ki67 in Breast Cancer Working Group, the validity of Ki67 immunohistochemistry as a prognostic marker in the clinical practice of breast cancer has been verified, especially for the identification of oestrogen receptor‐positive and HER2‐negative patients who do not need neoadjuvant therapy.^6^ For neuroendocrine neoplasms, the newest guideline from the WHO classification emphasizes the importance of Ki67 in classification and grading,^7^ and it has been determined to be an independent risk prognostic marker in high‐grade neuroendocrine tumours and carcinomas, which typically exhibit highly aggressive and advanced stages.^8^ Because of its high sensitivity, good specificity, flexible detection ability and reliability in prediction, Ki67 has been accepted as an ideal biomarker for prognostic assessment in the clinical setting of breast and neuroendocrine cancers; however, whether Ki67 is a prognostic marker in NSCLC remains to be elucidated.

Although Ki67 has received attention as a latent prognostic indicator in NSCLC, several studies have found that high Ki67 expression leads to poor outcome,^9^, ^10^ indicating its potential as an appealing prognostic biomarker in NSCLC. However, in clinical practice, the prognostic prediction of Ki67 in NSCLC has not achieved satisfactory consistency. Lung adenocarcinoma (LUAD) and lung squamous carcinoma (LUSC) are the most common histological subtypes of NSCLC and have emerged to show significant differences in biological behaviour, therapeutic targets and the immune microenvironment. In fact, previous meta‐analyses were not performed separately for different histological types of NSCLC, and the results in different subgroups did not agree, possibly suggesting that the insufficiently robust prognostic performance of Ki67 observed in the clinical practice of NSCLC might be associated with the biological heterogeneity of different histological types. Furthermore, as a reflector of active proliferation, the differentiated prognostic performance of Ki67 in NSCLC may be related to the heterogeneity in proliferative activity and the resulting differences in the biological behaviour of tumours; thus, an integrated analysis of tumour growth, metastatic risk and microenvironment would also help to explain the enigma of prognostic heterogeneity of Ki67 in NSCLC.

To clarify this issue, we systematically assessed the prognostic implications of Ki67 protein expression separately in LUAD and LUSC through meta‐analyses of published literature and survival analysis of a retrospective cohort from our center. In addition, publicly available transcriptome datasets were collected to evaluate the prognostic performance of Ki67 at the mRNA level and to study the factors responsible for the prognostic differences in Ki67 expression between LUAD and LUSC based on the analysis of tumour proliferation.

## 2 MATERIALS AND METHODS

### 2.1 Preparation of datasets

**FIGURE 1:**
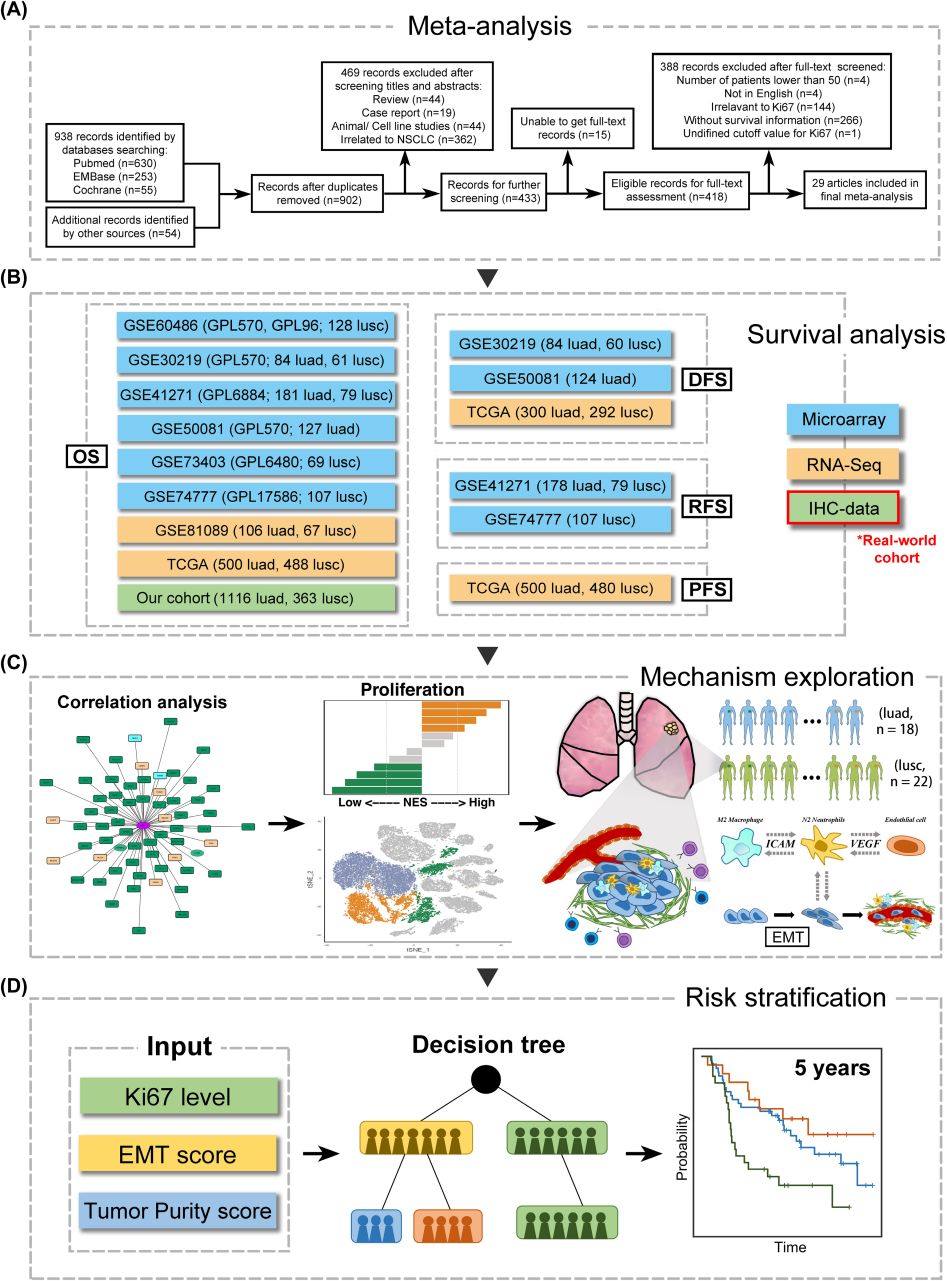
Flow chart of the study design.

According to the detailed strategies in Appendix S1 and Figure 1A, we searched all literature in PubMed, Embase and the Cochrane Library up to 2019. Eligible studies were included in the meta‐analysis to assess the prognostic value of Ki67 protein expression detected by immunohistochemistry (IHC) in LUAD and LUSC. A total of 1116 LUAD and 363 LUSC patients who had treatment records at the First Hospital of China Medical University and had overall survival information from November 2009 to the last follow‐up completed before February 2019 were included in our real‐world cohort. Among them, 43.41% of patients were in Stages I‐IIIA and 55.85% were in stage IIIB‐IV at diagnosis. The Ki67 expression values of patients were retrospectively obtained from IHC reports of diagnostic lung cancer surgical or biopsy specimens, and the percentage of tumour cells positive for Ki67 protein was used to estimate the Ki67 expression value. Based on previous studies,^11^, ^12^ patients with positive Ki67 protein expression in >25% of tumour cells were defined as having high Ki67 expression in our cohort. Eight public‐access datasets of microarray or RNA‐Seq data with totally 998 LUAD and 999 LUSC patients which had available survival information and the Ki67 expression acquired from surgically treated tumour tissue without neoadjuvant treatment were downloaded. We also acquired a single‐cell trasnscriptional dataset which included 18 LUAD and 22 LUSC patients for tumour microenvironment analysis. Seurat R package was used for preprocessing the single‐cell dataset that low‐quality cells with either gene number more than 5000 or fewer than 200, or mitochondrial gene accounting over 30%, were excluded before downstream analysis. Batch‐correction of merged samples was done by harmony R package. The study was approved by the Ethics Committee of the First Affiliated Hospital of China Medical University. Details are provided in the Appendix S1.

### 2.2 Comparison of proliferative subgroups

Based on four proliferative gene sets from MsigDB, gene set variation analysis (GSVA) and UCell method were used to score the proliferative activity of patients and epithelial cells at bulk and single‐epithelium‐cell transcriptome level respectively,^13^, ^14^ and patients and epiehtlial cells were grouped into the high proliferative group (proliferative score ≥0.1), low proliferative group (proliferative score ≤−0.1) and median group (−0.1≤ proliferative score ≤0.1) as described in a previous study.^15^ Gene set enrichment analysis (GSEA) and survival analyses were conducted between the different proliferative groups. The pathifier algorithm was applied to assess the degree of proliferation dysregulation mediated by the expression of Ki67 and related genes in tumours compared to normal tissues.^16^ Pearson correlation analysis was used to compare the relationship between proliferation scores, expression of Ki67 and 35 known epithelial‐mesenchymal transition (EMT) promoting genes. The expression of genes identified in the tumour transition states during EMT^17^ were evaluated in the epithelial cells of different proliferative groups. Monocle2 R package was used to build the single‐cell pseudotime trajectory and to quantify the proliferation and EMT status of malignant epithelium along the evolution^18^ (details are provided in the Appendix S1).

### 2.3 Assessment of tumour microenvironment

ESTIMATE algorithm was used to evaluate the infiltration of immune and stromal component in different proliferative subgroups.^19^ The infiltrative levels of specific immune cells and the representative stromal components, including CAFs and endothelial cells, were predicted using the deconvolution and ssGSEA algorithms based on the collected signatures.^20^, ^21^, ^22^ The enriched proportions of immune cells in each prolferative group were calculated by a pre‐ranked GSEA method with the enriched criteria of FDR adjusted *p* < 0.1 and NES >0. Based on cell markers, we identified the cell types in the single‐cell dataset, and the proportions of each cell component in patients of different evolutionary tracks were compared. The cell–cell interative network was built by cellchat toolkit, which was also used to analyse the direction and the strength of communication between cells.^23^

### 2.4 Risk stratification

After integrating the potential risk factors, patients were classified by survival tree analysis into different risk groups,^24^ which has been verified to be effective for the risk stratification of patients in recent studies on pancreatic ductal adenocarcinoma.^25^, ^26^ Then, a nomogram model was established, and the performance of survival prediction was evaluated using the *c*‐index and ROC curve (details are provided in the Appendix S1).

### 2.5 Statistical analysis

Survival analysis was performed using the log‐rank test, landmark analysis, univariable and multivariable Cox regression, and Student's *t*‐test, Wilcoxon rank sum test and one‐way ANOVA were used to compare groups. Pearson's correlation analysis was used to reveal the relationships between the genes and pathways. All analyses were performed using R version 4.0.1. All hypothetical tests were bilateral, with *p* < 0.05, indicating statistical significance.

## 3 RESULTS

### 3.1 The prognostic value of Ki67 protein expression in LUAD is the opposite of that in LUSC

In the meta‐analysis, 29 articles were included and were identified to have reputable quality with NOS scores ranging from 7 to 9 according to retrieval strategies. The characteristics of studies included in the meta‐analysis were displayed in Appendix S2. In the analysis of LUAD, the sample size of the studies varied from 52 to 482 (total: 4755, median: 140.5), and the median Ki67‐positivity was 16.5% (range: 5%–50%). Among the 22 studies and nine studies that analysed overall survival (OS, *I* ^2^ = 81%, *p* < 0.01) and disease‐free survival (DFS, *I* ^2^ = 65%, *p* < 0.01), patients with high Ki67 expression had a higher risk of death in LUAD (HR: 1.15, 95% CI: 1.07–1.23, *p* = 0.0001; HR: 2.12, 95% CI: 1.41–3.17, *p* = 0.0003; Figure S1A,C). For LUSC, the sample size of the studies varied from 81 to 437 (total: 1431, median: 119), and the median Ki67‐positivity was 30% (range: 5%–50%). Among the seven studies calculating OS (*I* ^2^ = 74%, *p* < 0.01), high Ki67 expression indicated a lower risk of death in LUSC (HR: 0.75, 95% CI: 0.65–0.88, *p* = 0.0003, Figure S1E). Cumulative and sensitivity meta‐analyses robustly supported the inverse prognosis of Ki67 protein expression in LUAD and LUSC (Figures S1B,D,F and S2A–C). For LUSC, the funnel plot and Egger's test showed no evidence of publication bias, indicating reliable results of the meta‐analysis (*p* = 0.7957; Figure S2F,I). However, the asymmetrical funnel plot reflected bias in the meta‐analysis of LUAD (Figure S2D,E), especially between the OS‐related studies (Egger's test, *p* < 0.0001). Although the meta‐analysis concluded that the DFS endpoint was significant after trim‐filling, more heterogeneities besides publication bias were found in OS‐related studies of Ki67 in LUAD (Figure S2G,H).

**FIGURE 2:**
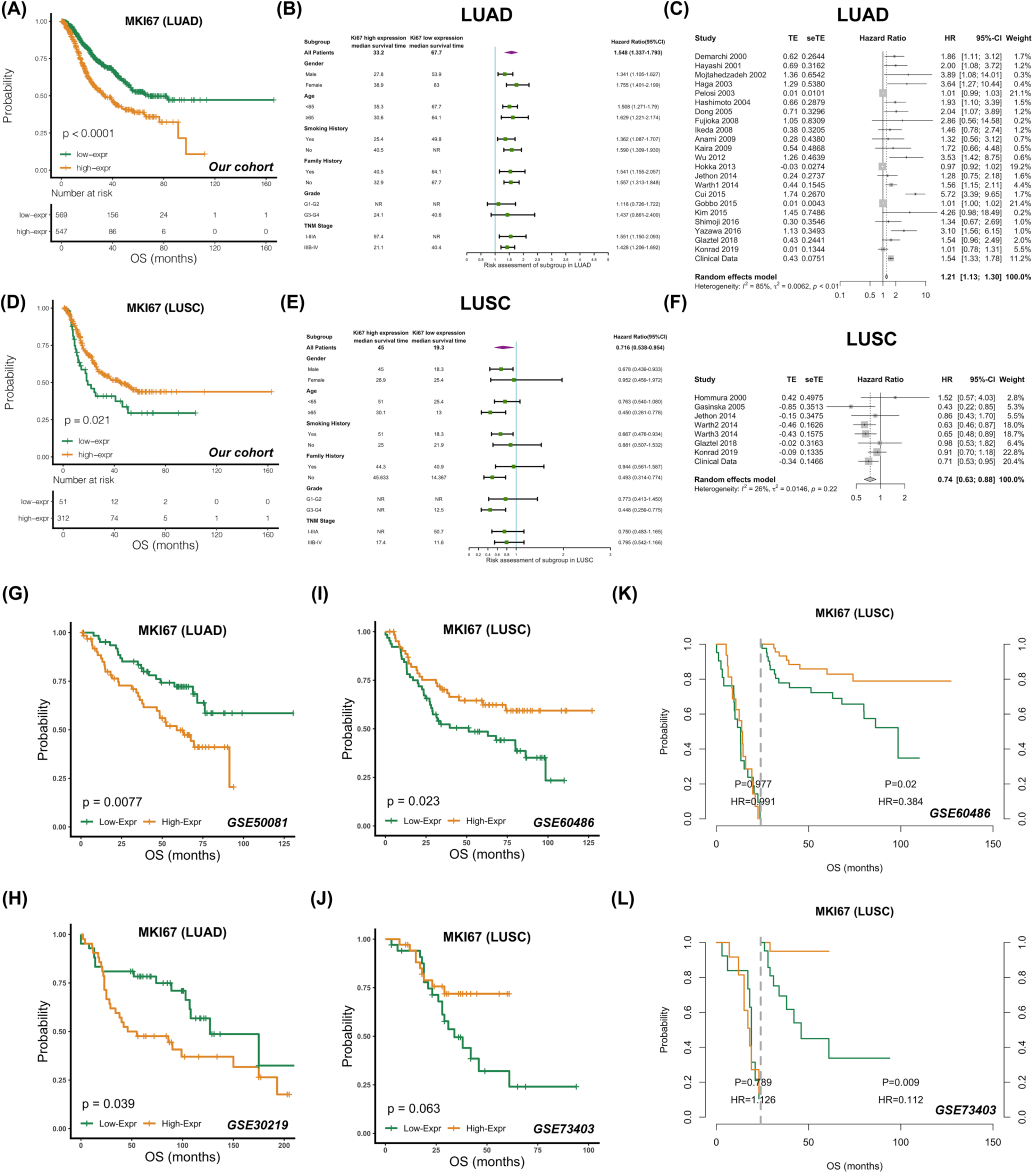
Prognostic evaluation of Ki67 in LUAD and LUSC with OS as the endpoint. (A, B) Prognostic analysis of Ki67 protein expression in (A) all and (B) sex, age, smoking history, family history, malignancy grade and pathological stage subgroups of LUAD patients in our cohort. ‘NR’ denotes unavailable data. (C) Meta‐analysis on our cohort and other published studies to evaluate prognostic value of Ki67 protein expression in LUAD. (D–F) The same analyses were performed in LUSC as in LUAD. (G, H) Prognostic analysis of Ki67 mRNA expression in LUAD patients from GSE50081 and GSE30219. (I–L) Prognostic analysis of Ki67 mRNA expression in LUSC patients from GSE60486 and GSE73403. The time point for landmark analysis was 24 month.

The findings from the meta‐analysis were further validated in our cohort, which included 1479 NSCLC patients (LUAD: 1116, LUSC: 363). Detailed clinicopathological characteristics of the patients are presented (Table S1) and Compared to LUAD, more LUSC patients had a history of smoking (78%) and were diagnosed at an earlier stage (62%), which is consistent with a previous large‐scale study of lung cancer.^27^ More patients with LUSC were diagnosed with high Ki67 expression under the same cut‐off, for which high Ki67 protein expression was defined as expression in more than 25% of tumour cells, which was in agreement with previous research, suggesting that the mean expression of Ki67 was higher in LUSC than in LUAD.^28^, ^29^ and the prognostic value of Ki67 was significantly reverse in LUAD and LUSC under the cut‐off (Figure 2A,D). In univariate analysis with OS as the endpoint, high Ki67 expression indicated poor outcome in LUAD (HR: 1.548, 95% CI: 1.337–1.793, *p* < 0.05), but conversely, low Ki67 expression suggested poorer survival in LUSC (HR: 0.716, 95% CI: 0.538–0.954, *p* < 0.05) and reverse prognostic value of Ki67 between LUAD and LUSC showed consistent tendency across multiple subgroups (Figure 2B,E). After adding our cohort, the conclusion of the meta‐analysis remained unchanged as being significantly opposite to the prognostic role of Ki67 between LUAD and LUSC (HR: 1.21, 95% CI: 1.13–1.30, *p* < 0.0001; HR: 0.74, 95% CI: 0.63–0.88, *p* = 0.0005, Figure 2C,F). In the multivariate Cox regression analysis, Ki67 was identified as an independent risk factor for LUAD (HR: 1.384, 95% CI: 1.191–1.609, *p* < 0.001, Table S2), and there was certainty that Ki67 was a favourable prognostic indicator in LUSC (HR: 0.76, 95% CI: 0.57–01.015, *p* = 0.063, Table S3). We subsequently evaluated the prognostic value of Ki67 using other pathologically accepted cut‐off values. As a poor prognostic factor, the conclusions were steady and cut‐off insensitive in LUAD (Figure S4A), whereas in LUSC, the prognostic role of Ki67 was insignificant but tended to be protective under most cut‐off values (Figure S4B). We also found a significant association between high Ki67 expression and advanced disease (stage IIIB‐IV; *p* < 0.001) in LUAD, whereas patients with low Ki67 expression were more likely to be linked to distant metastasis (stage IV; *p* = 0.007) in LUSC, reinforcing the notion that Ki67 had different prognoses in LUAD and LUSC.

Taken together, the opposite prognostic performances of Ki67 in different histological types of NSCLC both in meta‐analysis and in our cohort were observed; Ki67 was an unfavourable prognostic factor in LUAD, but conversely, low Ki67 protein expression indicated worse prognosis in LUSC. Moreover, our cohort had a discernibly higher ratio of patients with advanced‐stage LUSC (Figure S4C,D), extending the population to which conclusions applied that the prognostic performance of Ki67 at IHC level was consistent in each stage.

### 3.2 Low Ki67 mRNA expression predicts poor prognosis in LUSC after long‐term survival

Six microarray datasets and two RNA‐Seq datasets were collected to explore the prognostic heterogeneity of Ki67 expression between LUAD and LUSC at the transcriptional level. Since the TCGA cohort consisted of a larger number of patients, patients were divided into high, medium and low Ki67 expression subgroups according to the ⅓percentile to enable further patient stratification. For the remaining datasets, the median expression of Ki67 was used as the cut‐off value for dichotomy. In the analysis with OS as the endpoint, Ki67 was a distinct marker of poor survival in LUAD (Figures 2G,H and Figure S3A–C) but the prognostic value of Ki67 expression in LUSC was vague. Notably, the survival curves crossed in most LUSC datasets, suggesting that a log‐rank test might be inappropriate (Figures 2I,J, and Figure S3D,F,H,J,L). After correction by landmark analysis, we observed in LUSC that those with lower Ki67 mRNA expression at diagnosis had a poorer prognosis after at least 2 years of follow‐up (range between datasets: 24–72 months; median: 51 months), whereas before that, the mRNA expression level of Ki67 was not associated with prognostic differences. The results were significant in GSE60486, GSE73403, GSE30219 and GSE74777 (Figures 2K,L and Figure S3G,I), whereas other datasets showed a trend without reaching statistical significance (Figure S3E,K,M).

Subsequently, survival and landmark analyses in LUSC datasets were conducted using DFS, RFS and PFS as endpoints (Table S4). In the RFS analysis, landmark analysis could not be performed in GSE41271 given the paucity of mortality events after 48 months, while the result was insignificant in GSE74777. As for the analysis of DFS or PFS, using 5‐year follow‐up as the time point (range between datasets: 48–72 months; median: 64 months), the conclusions were consistent with those of studies on OS, indicating that Ki67 mRNA expression shared a common prognostic pattern in predicting OS, DFS and PFS.

Interestingly, meta‐analysis of four squamous carcinomas originating from the oesophagus, head and neck, lung and cervix in the TCGA dataset showed low Ki67 mRNA expression, indicating worse OS (HR = 0.83, *p* = 0.0381), while in the additional 14 types of solid tumours, the prognostic value of Ki67 was converse to that in squamous carcinoma (HR = 1.46, *p* = 0.0013). The prognostic differences reflected in the histological heterogeneity of squamous and non‐squamous carcinoma further support that the prognostic value of Ki67 should be evaluated separately in LUAD and LUSC.

### 3.3 Ki67 can reflect the heterogeneous prognostic role of tumour proliferation between LUAD and LUSC

**FIGURE 3:**
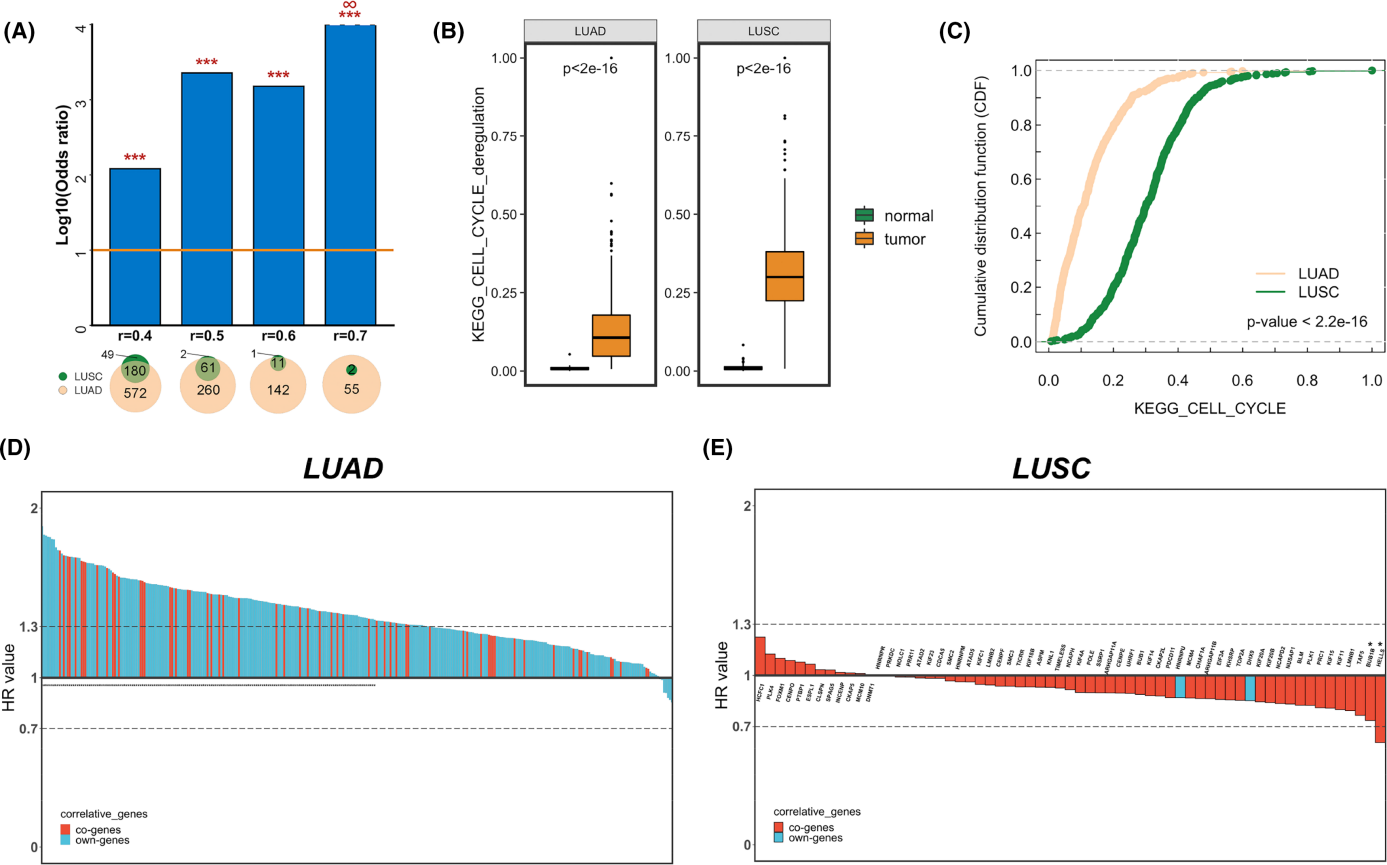
Relationship between Ki67 and proliferative activity in the TCGA cohort. (A) Calculation of the numbers of overlapping Ki67 correlated genes between LUAD and LUSC by Pearson correlation tests (*r* ≥ 0.4/0.5/0.6/0.7, *p* < 0.001) and a two‐side hypergeometric test (***p < 0.0001). (B) Dysregulated degree of KEGG_CELL_CYCLE pathway reflected by the expression of Ki67 and the correlated genes (*r* ≥ 0.5, *p* < 0.001). (C) Comparison of KEGG_CELL_CYCLE pathway dysregulated scores between LUAD and LUSC by Kolmogorov–Smirnov test. (D, E) Prognostic value of Ki67 correlated genes (*r* ≥ 0.5; *p* < 0.001) in LUAD (D) and LUSC (E) calculated by a log‐rank test (*p < 0.05).

Based on the GSEA of HALLMARK, KEGG and GO‐BP gene sets, the main biological function of Ki67 in NSCLC was confirmed to promote proliferation (Table S5). Four accepted proliferative gene sets, with diverse genes and gene numbers (Figure S5B) were collected to further compare the relationship between Ki67 expression and proliferative activity in LUAD and LUSC. Ki67 expression was significantly and positively correlated with the enrichment scores of these pathways and displayed a higher correlation in LUAD (Figure S5A). There was a significant overlap of Ki67 correlated genes between LUAD and LUSC in the TCGA cohort, as evidenced by Fisher's exact test, and the number of Ki67 correlated genes was much higher in LUAD (Figure 3A), with more intersected genes between Ki67 correlated genes and proliferative gene‐sets in LUAD than in LUSC (Figure S5B), suggesting that Ki67 is more closely associated with the proliferative activity in LUAD. However, Ki67 caused significant dysregulation of proliferation in tumour tissues compared to that in normal tissues (Figure 3B and Figure S5C,E), which was more severe in LUSC (Figure 3C and Figure S5D,F), indicating that LUSC grew faster than LUAD did.

**FIGURE 4:**
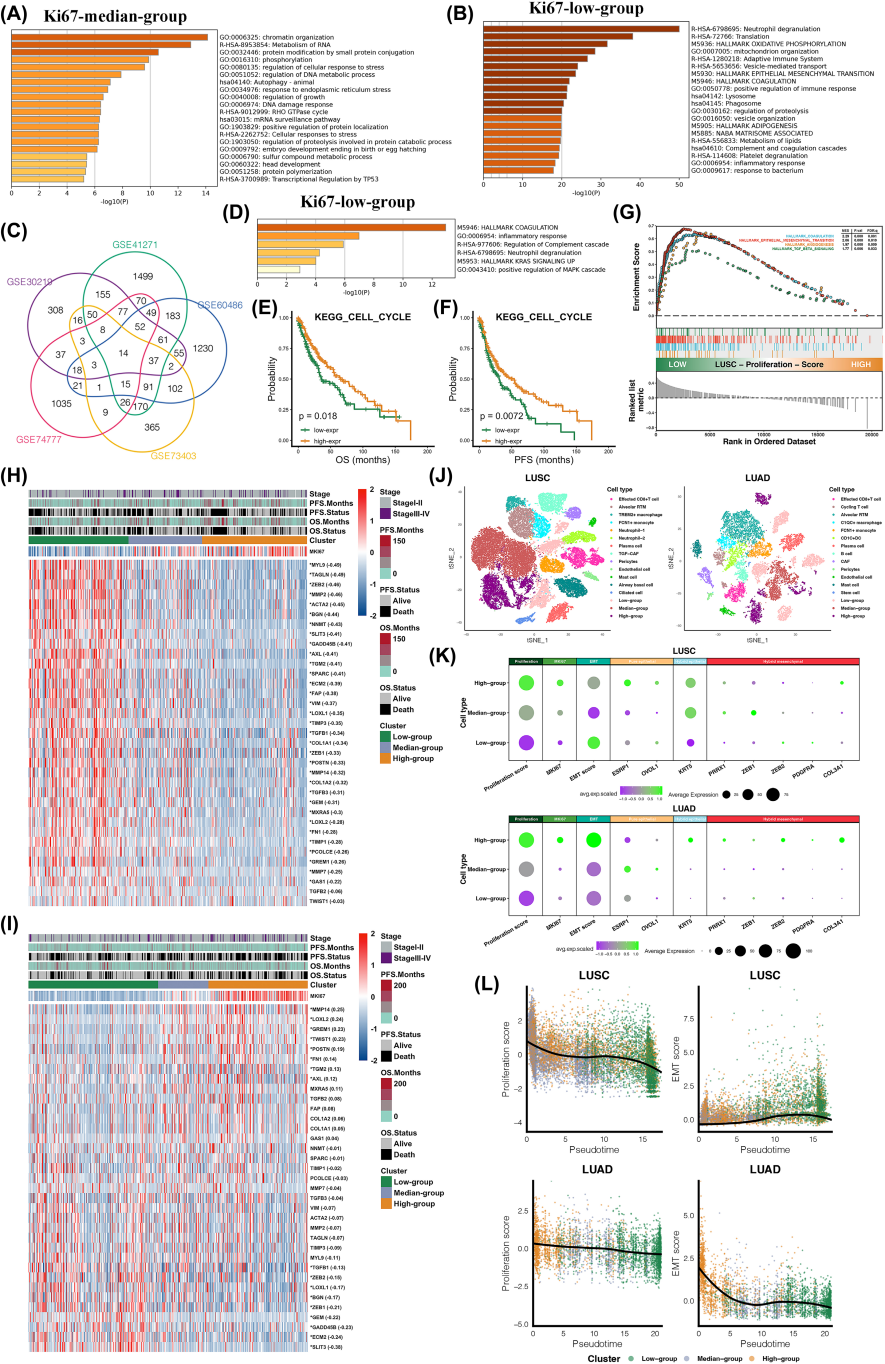
Relationship between EMT potential and the proliferative activity. (A, B) Significantly enriched pathways of median (A) and low (B) Ki67‐expression group in TCGA‐LUSC. (C) Venn plot of the differentially expressed genes of low Ki67‐expression group in microarray datasets. (D) Significantly enriched pathways of the shared differentially expressed genes of low Ki67‐expression group in microarray datasets. (E, F) Prognostic comparison of subgroups classified by KEGG_CELL_CYCLE proliferative scores in TCGA‐LUSC with (E) OS and (F) PFS as the endpoint. (G) Significant enrichment of low proliferative group in TCGA‐LUSC based on the hallmark gene‐set. (H, I) Pearson correlation test between the expression of Ki67 and 35 EMT‐promoting genes in (H) TCGA‐LUSC and (I) TCGA‐LUAD (*p < 0.01). (J) TSNE visualization of LUSC and LUAD cell clusters in the scRNA‐Seq dataset GSE148071. (K) Comparing the average expression of proliferation score, Ki67, EMT score and markers of EMT process between cancer cells of different proliferative groups in LUSC and LUAD. (L) Dynamic curves of proliferation score and EMT score along the developmental pathway of LUSC and LUAD tumour cells from high to low proliferation potential.

According to the log‐rank test, 97.8% of Ki67 correlated genes (*r* > 0.5, *p* < 0.001) indicated poor survival in LUAD, with 54.1% of them reaching statistical significance, whereas in LUSC, most of the same Ki67 correlated genes were supposed to be protective factors for prognosis (HR <0; Figure 3D,E). Since the opposite prognostic tendency of Ki67 and its highly correlated genes, it is reasonable to envisage that proliferative activity may also exhibit prognostic differences between LUAD and LUSC. In TCGA, analysis using OS and PFS as endpoints indicated that high proliferation was associated with poor survival in LUAD (Figure S6A,B,D–L). However, there was a higher prognostic risk of low proliferation in LUSC (Figures 4A,B, and Figure S7A–I). No prognostic differences were found in the analysis of DFS between LUAD and LUSC (Figure S6C). Validations were performed on GSE30219 and GSE81089 based on the KEGG_CELL_CYCLE enrichment score. Among LUAD patients in GSE30219, a microarray dataset, prognosis was significantly poorer in the high‐proliferation group, as reflected in OS and DFS (Figure S9C,D). For LUSC, the prognostic value of proliferative activity was similar to that of Ki67; low proliferation showed a tendency toward poorer prognosis in long‐term follow‐up after the crossing of survival curves (Figure S9A,B). The conclusions of GSE81089, an RNA‐Seq dataset containing only OS information (Figure S10A,B) were consistent with GSE30219. Thus, a close relationship was observed between Ki67 and proliferative activity, and Ki67 could reflect the heterogeneous prognostic role of tumour proliferation in both LUAD and LUSC.

### 3.4 Low Ki67 expression corresponds to higher EMT potential in LUSC

**FIGURE 5:**
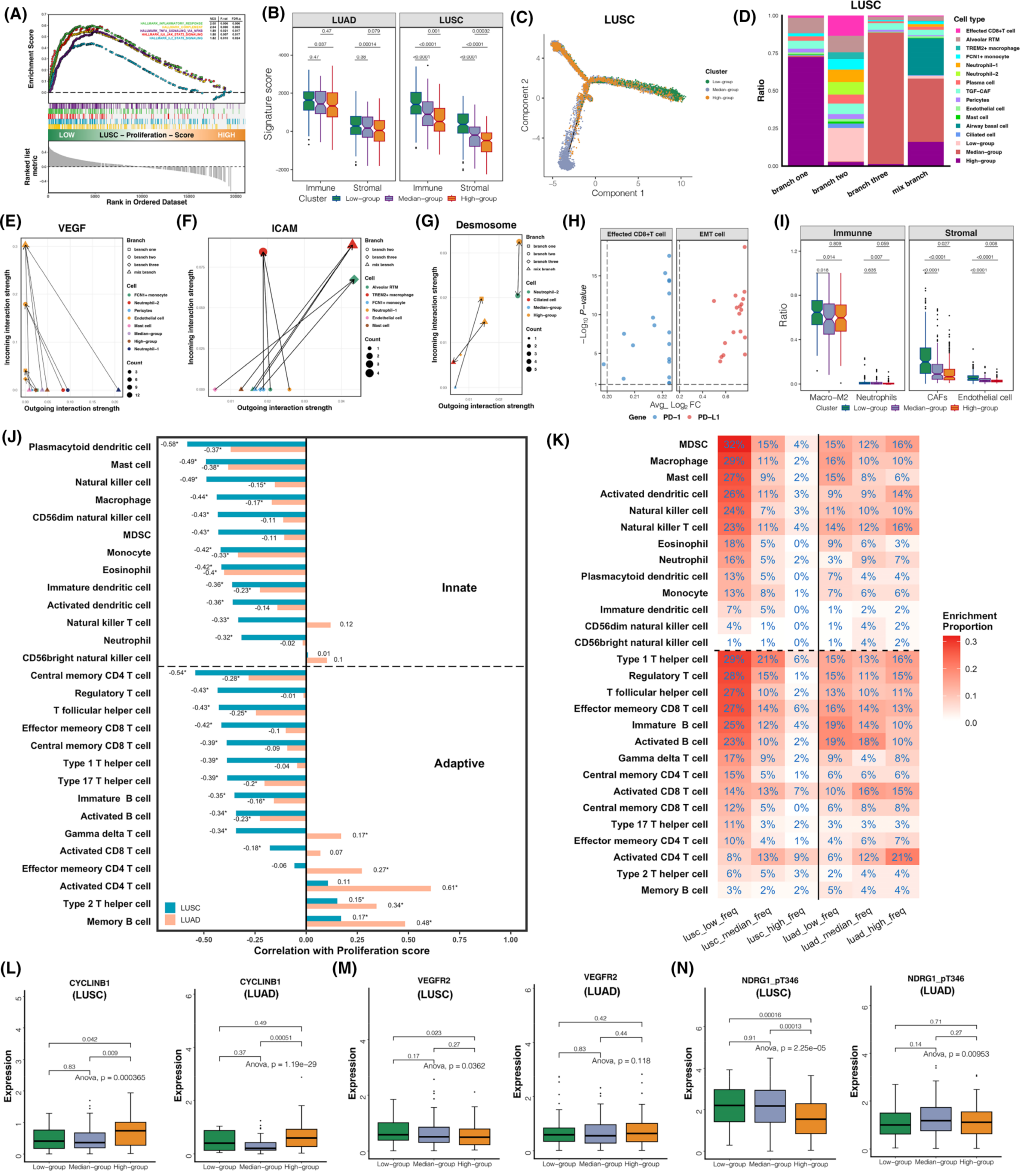
Patterns of tumour microenvironment among different proliferative subgroups. (A) Significant enrichment of immune‐associated pathways in the low proliferative group of TCGA‐LUSC based on the hallmark gene set. (B) Wilcoxon test of immune score and stromal score between patients of different proliferative group in TCGA‐LUSC and TCGA‐LUAD. (C) Differentiation trajectory of LUSC cancer cells grouped by proliferative capacity in the scRNA‐Seq dataset. (D) Cell proportion of LUSC tumours of different trajectories in the scRNA‐Seq dataset (branch one: *n* = 2, branch two: *n* = 12, branch three: *n* = 2, mix branch: *n* = 6). (E–G) Intercellular communications of (E) VEGF, (F) ICAM and (G) DESMOSOME signalings from outgoing cells towards incoming cells calculated by Cellchat in LUSC tumours. Axis represents the outgoing or incoming communication probability associated with each cell group. Dot size is proportional to the size of inferred links. Dot shape corresponds to tumours of different branch and dot colour represents the cell types. (H) Expression comparison of PD‐1 between effected CD8 + T cells and other cell type, and of PD‐L1 between EMT cells and other cell type in the low‐proliferating LUSC tumours, which was computed by the MAST algorithm of FindMarkers function of Seurat. The dashed lines correspond to 0.2 on the x‐axis and 1 on the y‐axis. (I) Wilcoxon test of immune cells in different proliferating groups in TCGA‐LUSC, including the proportion of M2‐macrophage in all macrophages and neutrophils, as well as stromal cells including CAFs and endothelial cells. (J) Pearson correlation analysis between KEGG_CELL_CYCLE proliferative scores and immune cell enrichment scores in LUAD and LUSC (*p < 0.001). (K) The proportion of patients with significant infiltration of specific immune cells in each proliferative subgroup of LUAD and LUSC. (L–N) The protein level of (L) CYCLINB1, (M) VEGFR2 and (N) NDRG1_pT346 in different Ki67‐expression group in the TCPA‐LUSC and TCPA‐LUAD database.

We conducted pathway enrichment analysis on differentially expressed genes (Log_2_FC >0.25 and *p* < 0.05) among different Ki67 expression groups within the TCGA‐LUSC dataset. As we expected, the group with high Ki67 expression predominantly corresponded to activation of proliferation pathways (Table S5). Pathways related to nucleic acid metabolism and DNA damage repair were enriched in the group of median Ki67 expression with better prognosis (Figure 4A). Interestingly, pathways associated with epithelial‐mesenchymal transition, tumour coagulation and positive immune regulation were significantly enriched in the low Ki67 expression group of poorer prognosis (Figure 4B). We identified 14 genes that were commonly upregulated in patients with low KI67 expression across multiple LUSC microarray datasets with the screening critia: Log_2_FC >0.25 and *p* < 0.05 (Figure 4C). These intersecting genes were mainly functioned in pathways of coagulation and immune response, which were consistent with the findings from TCGA (Figure 4D). Low‐proliferation tumours in LUSC also exhibited poorer prognosis (Figure 4E,F) and pathways associated with tumour metastasis, including coagulation, EMT, angiogenesis and TGF‐β, were noticeably enriched in the low‐proliferation group of LUSC but were not detected in LUAD (Figure 4G). Then, 35 recognized EMT‐promoting genes were collected to verify the results of GSEA. In LUSC, most EMT‐promoting genes had higher expression in the low proliferative group and were significantly negatively correlated with the expression of Ki67 (Figure 4H), which were also obserbed in GSE30219 (Figure S9E), GSE81089 (Figure S10C) and CHOICE cohort (Figure S11A). However, no specific expressive pattern was found between the EMT‐promoting genes and Ki67 in LUAD (Figure 4I, Figures S9F, S10D and S11B). We further verified at the level of single‐cell that the relation of EMT and tumour proliferation was different between LUAD and LUSC. A total of 13,724 cells of 24 cell types from 18 LUAD patients and 40,835 cells of 22 cell types from 22 LUSC patients were identified through quality control and batch correction, and the correctness of cell types annotation was proved by the marker genes (Figure S8A,B). Similarly, as with bulk RNA‐Seq for tumour samples, malignant epithelial cells were divided into high, median and low proliferating cell groups based on proliferation enrichment scores in single‐cell analysis (Figure 4J). It was surprised to see that in tumour cells of LUAD, high expression of Ki67 corresponded to stronger EMT potency, while in LUSC, low proliferating malignant epithelium were more likely to occur EMT (Figure 4K). We additionally characteried the relationship between tumour proliferation and EMT by evaluating the dynamic process of EMT. Referring to previous study, the expression of epithelial markers was highest in high‐proliferating cell population of LUSC, and then in the development from hybrid epithelial state to hybrid mesenchymal state, EMT process‐related marker genes were increasingly expressed in median and low proliferation tumour cell groups (Figure 4L). However, compared to epithelial markers, hybrid epithelial and hybrid mesenchymal markers were more highly expressed in LUAD tumour cells with high Ki67 expression, indicating a greater likelihood of EMT. Based on the evolutionary trajectory constructed from high to low proliferation cell populations (Figure 5C and Figure S8C), it was found that EMT had an upward trend along the pseudotime in LUSC, but descended in LUAD as tumour proliferation capacity declined (Figure 4L). In conclusion, high EMT level is one of the potential factors that made low Ki67 expression suggested poor prognosis in LUSC.

### 3.5 Less proliferative tumours in LUSC were characterized by immunosuppressive microenvironment

Several inflammation‐related pathways were also enriched in the low‐proliferation group of TCGA‐LUSC dataset (Figure 5A). The differences of immune and stromal infiltration among proliferation groups were more prominent in LUSC than in LUAD, and the tumour microenvironment with low proliferative activity were significantly ‘hotter’ in LUSC (Figure 5B). At single‐cell level, three trajectories of cancer cells showing high, low and median proliferation capacity were formed in the evolution of LUSC, named branch one, two and three (Figure 5C). According to the major differentiation direction, which was defined as the proportion of tumour cells in a certain trajectory exceeded 1/3, LUSC patients were also divided into branch one (*n* = 2), two (*n* = 12), three (*n* = 2) and mixed branch group (*n* = 6). For tumours dominated by high proliferating epithelium, immune and stromal cells infiltrated only a small amount (less than 25%) in the tumour microenvironment. In contrast, tumours that evolved towards low‐proliferation branching two, consisting mostly of non‐epithelial cells (about 75%), corresponding to a ‘hotter’ tumour microenvironment in consistent with the result of bulk RNA‐Seq (Figure 5D). We noticed that almost all the tumour‐associated‐neutrophils (TAN), including neutrophil‐1 and neutrophil‐2, were presented in the tumours of branch two, and TREM2+macrophage and endothelial cell also had a higher proportion of infiltration comparing to that in branch one and three. Both neutrophil‐1 and neutrophil‐2 were identified as N2‐TAN based on upregulated expression of STAT3, which promoted N2 polarization of neutrophils^30^ (Figure S8A). As an important mediator of angiogenesis,^31^ neutrophils were found to be the major source of VEGF communicating toward endothelial cells in the branching‐two tumours (Figure 5E). In neutrophil‐1, overexpression of HIF1A enhanced VEGF expression in response to hypoxia,^32^ and the activation of CSF3R‐NAMPT‐STAT3 signalling augmented angiogenesis and tumour growth.^33^ The JAK‐STAT3 signalling pathway activated by GM‐CSF increased ICAM1 expression, that facilitated tumour metastasis through neovascularization.^34^ High expression of LCN2 in neutrophil‐2 positively regulated the expression of VEGF to stimulate angiogenesis,^35^ and CEACAM6 induced neutrophil‐2 to adhere to the endothelial cells, further involving in angiogenesis and tumour progression.^36^ In hypoxic microenvironment, neutrophils of branching‐two tumours were closely related to macrophages, which not only encouraged macrophage to convert to M2‐TAM phenotype,^37^, ^38^ but mediated adhesion and crosstalk with macrophage through ICAM‐1 (Figure 5F), and elevated expression of ICAM‐1 in macrophages had been reported to be associated with more aggressive tumours.^39^ Besides close communication with endothelial cells and macrophages, neutrophils from the branching‐two tumours integrated with neighbouring tumour cells by desmosomal junctions (Figure 5G), which helped tumour cells colonization and extravasation during tumour metastasis. STAT3 activated LCN2 secreted by neutrophils could also contribute to EMT, invasion and migration of tumour cells.^30^ PD‐L1 had the highest expression on EMT cells, that in addition to promoting the development of EMT in malignant epithelium, PD‐L1 imparted a potential immune suppressive character to cancer cells by binding to PD‐1 on CD8+T lymphocytes, expecially under hypoxic tumour microenvironment^40^, ^41^ (Figure 5H). However, no neutrophil cluster was identified in LUAD tumours (Figure S8B), and difference in the composition of microenvironment was not obvious among branches of LUAD tumours with different proliferative activity (Figure S8C,D). Findings of single‐cell analysis was confirmed in the TCGA‐LUSC dataset, that tumours of low proliferation encompassed significant higher ratio of M2‐macrophages, neutrophils, CAFs and endothelial cells than high proliferative tumours (Figure 5I). However, in LUAD, tumours in the high‐proliferative group had the highest proportion of M2 macrophage and more CAF infiltration, while infiltrative ratio of neutrophil did not differ significantly among proliferation groups (Figure S8E). Infiltrative score calculatd by ssGSEA algorithm of immunosuppressive components including macrophages, MDSC, neutrophils and regulatory T cells presented significantly negative association with proliferation score in LUSC tumours, but no noteworthy correlation with proliferative capacity was noticed in LUAD tumours (Figure 5J). We then quantified the proportion of patients enriched with certain immune cells in different proliferation subgroups and found that apparently more low‐proliferating LUSC tumours had significant enrichment of MDSC, macrophages, neutrophil and Tregs, while a lower proportion of high‐proliferating tumours showed appreciable infiltration of immune cells, which was consistent with the results of single‐cell analysis (Figure 5K). There was little difference in immunoinfiltration between proliferating subgroups in LUAD patients (Figure 5K). Results from TCGA were confirmed in other bulk datasets, including GSE30219 (Figure S9G–L), GSE81089 (Figure S10E–J) and the CHOICE cohort (Figure S11C–H). In TCPA‐LUSC and TCPA‐LUAD databases, we found that CYCLINB1 protein expression, a marker of proliferation, was higher in high‐Ki67 mRNA expression group than in low group, demonstrating the positive relationship between Ki67 mRNA expression and tumour proliferation activity at protein level (Figure 5L). The protein level expressions of VEGFR2 and NDRG1, the markers associated with angiogenesis and tumour metastasis, were significantly upregulated in low‐Ki67 expression group compared to high‐Ki67 expression in the TCPA‐LUSC database (*p* < 0.05). However, the expression of VEGFR2 and NDRG1 protein in the TCPA‐LUAD database did not show significant differences among different Ki67 expression groups (Figure 5M,N). Taken together, based on transcriptional analysis conducted in single‐cell and bulk levels, and verification at protein level, we unravelled that less proliferative tumour in LUSC was a complex assortment exhibited a higher EMT potential and an immunosuppressive microenviroment that was ‘hot’ but conductive to tumour angiogenesis and metastasis.

### 3.6 Construction of a prognostic model based on Ki67, EMT score and purity score in LUSC

**FIGURE 6:**
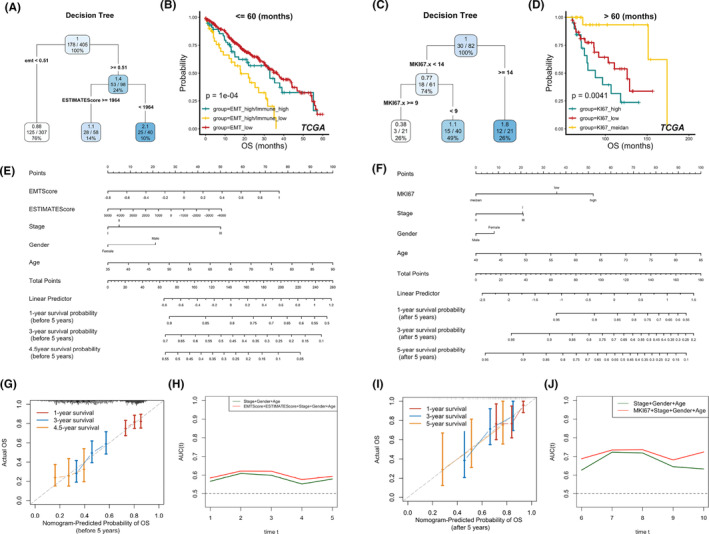
Risk stratification of patients in the TCGA‐LUSC cohort based on the expression of Ki67, EMT score and purity score. (A–D) Risk stratification and prognostic comparison of patients discriminated by survival tree analysis with OS occurring before (A, B) and after 5 years (C, D). (E, F) Integrating clinical parameters, Ki67 mRNA expression, EMT score and purity score to construct a nomogram of patients with OS occurring before (E) and after 5 years (F). (G–J) Predictive accuracy of the nomogram and performance comparison between the nomogram and common clinical models of patients with OS occurring before (G, H) and after 5 years (I, J).

Owing to the strong relationship between Ki67 expression and proliferative activity, we speculated that in LUSC, the prognostic role of Ki67 may be related to EMT potential and immune infiltration pattern. To validate this conjecture, Ki67 mRNA expression, EMT score and purity score were integrated into the survival tree models. Long‐term survival is defined as survival beyond 5 years after diagnosis. In the analysis of TCGA with OS as an endpoint, during the first 5 years of survival, patients were mainly stratified by EMT scores and then by purity scores (Figure 6A); high EMT scores or a cold tumour microenvironment indicated a significantly poorer prognosis (Figure 6B). However, after 5 years of survival, Ki67 was an important factor reflecting prognosis (Figure 6C), and low Ki67 mRNA expression suggested a poorer outcome (Figure 6D), which matched the results of landmark analysis (Figure S3K). We further validated this finding using other datasets. Considering the heterogeneity between datasets and the shorter follow‐up period, prognostic stratification in GSE73403 and GSE81089 were estimated using a 2.5‐year cut‐off. The conclusion obtained from TCGA was well validated in GSE30219, GSE74777, GSE60486, GSE41271, GSE73403 and GSE81089 that low Ki67 mRNA expression indicated poor prognosis in LUSC after long‐term survival (Figure S12). In the analyses using DFS and PFS as endpoints, the results obtained from GSE30219 and TCGA were similar to those obtained using OS. This indicated that the conclusion was robust across the different survival endpoints (Figure S13).

The potential risk factors acquired from survival tree analysis in the TCGA cohort and basic clinical characteristics, including age, sex and pathological stage, were then evaluated to construct nomogram models for LUSC before and after 5‐years of survival (Figure 6E,F), which accurately predicted the actual OS at 1, 3 and 5 years (Figure 6G,I). Furthermore, according to the analysis of time‐dependent ROC, the inclusion of EMT scores, purity scores and Ki67 mRNA expression levels could improve the predictive power of the basic model before and after 5‐years of survival, respectively (Figure 6H,J). Thus, nomograms constructed based on Ki67 gene expression could further complement the prediction of Ki67 protein expression levels in prognostic stratification of LUSC.

## 4 DISCUSSION

Recently, significant heterogeneity between LUAD and LUSC in terms of clinical features has been illustrated,^42^ but it is uncertain whether histological heterogeneity affects the prognostic performance of Ki67 in NSCLC. Here, we observed the opposite prognostic role of Ki67 between LUAD and LUSC, that high Ki67 expression was a risk factor for LUAD, but low Ki67 expression indicated a poor outcome in LUSC. We proved that although Ki67 reflected tumour proliferation activity in both LUAD and LUSC, low Ki67 expression in LUSC was accompanied by a higher EMT potential and an immunosuppressive microenvironment that facilitated tumour metastasis. Finally, nomogram models constructed by integrating clinical risk factors and Ki67 gene expression exhibited accurate prognostic predictions for LUSC.

Evidence supporting low Ki67 expression as an unfavourable prognostic factor associated with increased EMT capacity has been reported in other cancer types. In our meta‐analysis, besides squamous type carcinoma, we found that Ki67 tended to be a protective factor for colorectal and stomach adenocarcinomas, independent of other types of adenocarcinomas. Indeed, in previous studies of colorectal cancer, tumour cells at the invasive margin were detected with lower Ki67 expression, and budding tumour cells exhibited EMT‐like phenotypes and lower Ki67 positivity compared to tumour bulk,^43^, ^44^ while highly proliferative tumours possessed fewer invasive subclones, corresponding to a lower risk of metastasis.^45^ In gastric adenocarcinoma, multiple EMT‐related proteins representing invasive properties were negatively correlated with Ki67 expression, such that low Ki67 protein expression was also an indication of dismal outcome.^46^

We noted that the prognostic value of Ki67 in LUSC were generally consistent between protein and transcription levels, but there were still differences. At protein level, Ki67 expression detected by IHC that directly reflected the proliferative state of tumour cells, while bulk samples was an integration of all cells in the tumours, thereby the transcriptional expression of Ki67 was influenced by cell components other than cancer cells in the tumour tissues. In exploring the factors that affected the prognostic performance of Ki67, we demonstrated under single‐cell resolution that in LUSC, cancer cells with low proliferative activity do have a higher EMT potential and more EMT cells were presented in tumour. Furthermore, in addition to the variation in EMT levels, the immunosuppressive microenvironment formed by abundant infiltrations and the close intercellular communications of N2‐neutrophils, M2‐macrophages, endothelial cells, CAFs, MDSCs and Tregs in LUSC of low‐proliferation also played an important role in promoting angiogenesis and inhibiting the antitumour effect of CD8+T cells, thus created a favourable condition for tumour cells to develop EMT and distant metastasis. We discovered that neutrophils located with cancer cells through desmosomal junctions and transmitted VEGF signalling to endothelial cells to promote angiogenesis, which was consistent with the recent researches that neutrophils played a crucial role in tumour development, not only by recruiting and stabilizing tumour cells, but also by participating in neovascularization to facilitate tumour progression.^47^, ^48^ ICAM‐1 was found to be an important regulator of angiogenesis and to accelerate the malignant process of tumour.^49^, ^50^ Moreover, hypoxia further enhanced the pro‐tumourigenic effect of neutrophils, that specific deletion of HIF‐1α in neutrophil could significantly reduce tumour burden accompanied by increase number and activity of antitumour immune cells.^51^ Through crosstalk with tumour cells and recruitment of immunosuppressive cells, CAFs stimulated angiogenesis and provided a scaffold for tumour invasion and metastasis.^52^, ^53^ Cancer cells recruited Tregs and MDSC to undermine the infiltration of effector T cells within tumours and induced apoptosis, as well as emboldened M2‐macrophage to secrete cytokines for angiogenesis, thus forming an immunosuppressive microenvironment and promoting escape.^54^ In LUSC, significant activation of coagulation pathways occurred in samples with low Ki67 expression. It has been reported that the activation of EMT mediated by ZEB1 could trigger a procoagulant state driving metastasis of circulating tumour cells (CTC).^55^ The hypercoagulability developed by CTCs can also protect CTCs in turbulent blood flow.^56^ Although the prognostic value of Ki67 is unclear, EMT, CTCs and hypercoagulability have been demonstrated as adverse prognostic biomarkers of NSCLC.^57^ Based on our findings and previous studies, we postulated that in LUSC, tumours with low proliferation were prone to develop EMT during early survival, leading to worse prognosis. For patients with longer survival, initially metastatic CTCs were protected in hypercoagulable blood and seeded to dormancy in the metastatic soil until activation, thus reflecting low Ki67 expression but a poor outcome after long‐term survival. To this end, analyses to collect CTCs paired with tumour samples are in our future plans to clarify the mechanism by which low Ki67 expression reflects poor prognosis in LUSC, especially for patients surviving longer.

The information provided by Ki67 expression may help prioritize treatment options for LUSC. The possibility of insidious micrometastases requires attention under the diagnosis of low Ki67 expression, and in this case, angiogenesis and immunosuppression targeting were deemed promising strategies.^58^, ^59^ Furthermore, the occurrence of EMT in NSCLC was detrimental to the enrichment of immunocompetent cells and would increase the release of immunosuppressive cytokines, such as TGF‐β^60^ to develop resistance to erlotinib target therapy through the TGFβ‐IL‐6 axis.^61^ The application of EGFR‐TKI has been reported to alleviate abnormal coagulation, leading to longer OS in progressive lung cancer.^62^ Thus, we propose that EGFR‐TKI might be prioritized for LUSC patients who have low Ki67 expression at diagnosis but progress to advanced disease after long‐term survival.

Compared to previous research, our study covered IHC, microarray, bulk and single‐cell RNA‐seq data with a sufficient sample size and multifaceted data format to understand the heterogeneous prognostic role of Ki67 in LUAD and LUSC. However, there remain certain limitations in this research: Due to the retrospective nature and undefined clinical gold standard in the evaluation of Ki67, the definition of cut‐off value differed between studies in meta‐analysis. Data directly indicating the quality of life were not collected or evaluated in our real‐world cohort. In landmark analysis, the time point selected for the event varied due to differences in baseline characteristics and follow‐up duration across datasets.

In conclusion, our research demonstrates the different prognostic roles of Ki67 in LUAD and LUSC at both the protein and mRNA levels. High Ki67 expression indicated poor survival in LUAD, whereas low Ki67 expression suggested poor outcome in LUSC, coupled with a stronger metastatic potential. We highlight Ki67 as a useful prognostic biomarker for NSCLC, but should be considered separately in LUAD and LUSC to achieve a more reliable prognostic assessment.

## AUTHOR CONTRIBUTIONS

**Yunpeng Liu:** Conceptualization (lead); supervision (equal); writing – review and editing (equal). **Yujing Yang:** Investigation (lead); methodology (lead); writing – original draft (equal). **Xinye Shao:** Methodology (lead); writing – original draft (equal). **Zhi Li:** Methodology (supporting). **Lingyun Zhang:** Data curation (equal); supervision (supporting). **Bowen Yang:** Methodology (supporting). **Bo Jin:** Data curation (equal); investigation (supporting); supervision (supporting). **Xuejun Hu:** Supervision (supporting). **Xiujuan Qu:** Resources (supporting); supervision (supporting). **Xiaofang Che:** Conceptualization (equal); project administration (lead); supervision (equal); writing – review and editing (equal).

## FUNDING INFORMATION

This study was supported by the National Natural Science Foundation of China (Grant No. 81972197), Technological Special Project of Liaoning Province of China (2019020176‐JH1/103), Key Research and Development Program of Liaoning Province (2018225060) and China Medical University High‐level Talent Introduction Support Project (Project No.36003).

## CONFLICT OF INTEREST STATEMENT

The authors declare no conflict of interest.

## Supporting information


Appendix S1.

Appendix S2.

## Data Availability

Expression profile data analysed in this study were obtained from The Cancer Genome Atlas Program (TCGA), figshare at https://doi.org/10.6084/m9.figshare.7306364.v1 and Gene Expression Omnibus (GEO) at GSE60486, GSE30219, GSE41271, GSE50081, GSE73403, GSE74777, GSE81089 and GSE148071. The clinical data of patients from the First Hospital of China Medical University in this study are not publicly available due to patient privacy requirements but are available upon reasonable request from the corresponding author.
