# Supplementary material for: Prognostic heterogeneity of Ki67 in non‐small cell lung cancer: A comprehensive reappraisal on immunohistochemistry and transcriptional data
Source: J Cell Mol Med. 2024 Jul 17;28(14):e18521. doi: 10.1111/jcmm.18521 (PMC11255407; doi:10.1111/jcmm.18521)

**SUPPLEMENTARY MATERIAL**

**Supplementary Methods**

**Study design**

The flow chart of the study to assess the prognostic value of Ki67 and the risk stratification of patients based on the latent risk factors in LUAD and LUSC was shown in supplementary Fig. S1. First, by sourcing articles from 3 databases, 29 eligible(1-29) articles estimating prognostic value of Ki67 on the base of IHC in NSCLC were selected in the meta-analysis. An IHC cohort from our center and other 7 public-access datasets containing survival information of patients with NSCLC were used to validate the association between Ki67 expression and clinical outcome under multiple endpoints. We further explored the potential mechanisms that contribute to the prognostic variation of Ki67 in patients with NSCLC from the perspective of EMT and tumor microenvironment at bulk and single-cell resolution. Finally, risk stratification of patients with LUSC before or after survival of 5 years was extrapolated by decision tree analysis and was quantified by nomogram models for clinical application.

**Meta-analysis**

According to Preferred Reporting Items for Systematic Reviews and Meta-Analyses (PRISMA), we searched for articles published ranging from 2000 to January 4, 2020 in PubMed, EMBASE and Cochrane Library databases. Search terms included “Lung Neoplasm[MeSH]”, “Carcinoma, Non-Small-Cell Lung[MeSH]”, “Ki-67 Antigen[MeSH]”, “survival”, “outcome”, “prognos*” were also complemented with relevant free-text terms and these were, if appropriate, combined with operators. We retained articles meet the criteria: (1) Studies investigating the prognostic role (focus on time-to-event outcome) of Ki67 expression in patients with LUAD or LUSC; (2) The expression of Ki67 was obtained from IHC; (3) HR value and 95% CI or P value of the prognostic analysis could be directly gained from the article without any incorrection or contradiction; (4) Only recent or the most complete studies would be selected if the author had published multiple articles with the same population. To embrace a comprehensive purview, references of the included studies were also supplemented. Details of the exclusive criteria are available in Fig. 1A.

The process of article selection and data extraction was carried out by three researchers. Two of them searched out the studies separately, while the third one was up to resolve the conflict between the two researchers and make a final judgement. The quality of the included literature was estimated by Newcastle-Ottawa (NOS) quality evaluation criteria. The total score is 9 points and a considering high-quality article should not less than 7 points. Any disagreement was come to consensus with a third reviewer. After article screening, data was collected by two researchers and verified by another member if inconsistence existed. Information such as first author, year of publication, country, sample size, gender, mean age, pathological type, TNM stage, Ki67 cut-off value, median follow-up, survival endpoint, HR and 95% CI (multivariate analysis would be selected while the HR values were both provided in univariate and multivariate analysis) were extracted and prepared for the meta-analysis.

For statistical-analysis, Q test and I^2^ statistics were used to determine non-accidental observed heterogeneity. When P>0.1 or I^2^<50%, the studied were considered to have acceptable heterogeneity and a fixed model was selected; otherwise, a random effect model was selected. The standard error of HR logarithm was calculated by inverse variance method according to the HR and 95% CI. In order to obviate the sensitivity of HR to study selection allocation bias in explanation of observed heterogeneity, sensitivity analyses were performed after sequentially precluding studies in turn. If the number of studies included in the meta-analysis was more than 10 that the funnel plot and Egger’s test were used to determine publication bias; otherwise, the trim-fill method was used to identify publication bias and can be regarded as a quantitative supplement to the funnel plot. If the likelihood of publication bias is considered high detected by Egger’s test, the stability of conclusion should be reassessed by the trim-fill method.

**Patient collection**

Patients enrolled in this study had diagnosis of primary bronchopulmonary tumor confirmed by surgery, biopsy, or tumor cytology test with pathological diagnosis of adenocarcinoma or squamous carcinoma. Complete medical records of patients were available for retrospective retrieval of clinicopathological features and relevant parameters, such as age, gender, smoking history, family history, pathological tumor-lymph node-metastasis staging and tumor grading. Information of the tumor staging and Ki67 value was extracted from the pathology report obtained at the time of definite diagnosis. The study endpoint was overall survival, and the follow-up was conducted through two physicians separately, with the last follow-up ending in February 2019. For patients who were lost to follow-up, the date of last hospitalization was defined as the final survival time point. In LUSC, four Patients whose Ki67 values were not sourced from pathological diagnosis and five patients whose survival status or follow-up time could not be clarified were excluded from the study. In LUAD, four patients whose survival status or follow-up time were ambiguous were excluded from the study. The information of patients with the same name were critically reviewed. We searched for all the medical records from November 2009 to January 2019 of the Department of Medical Oncology, The First Hospital of China Medical University, ultimately 1116 patients with lung adenocarcinoma and 363 patients with squamous lung carcinoma were eligible and included in the study.

**Public-access datasets**

TCGAbiolinks(30) was used to download level 3 RNA-Seq expression profiles and the corresponding clinical information of patients with LUAD and LUSC from TCGA. Outcome data including OS, DFS and PFS were acquired from TCGA Pan-cancer clinical data resource(31). Patients who had received neoadjuvant treatment before sequencing, with missing follow-up information or without clear histopathological diagnosis were excluded from the analysis. Another RNA-seq cohort named CHOICE which were all Chinese and treatment-naïve patients, including 131 LUAD samples and 114 LUSC samples was obtained from the repository (32). The abundance of each RNA-Seq expression data was normalized by fragment per kilobase per million (FPKM) method in further analysis. An RNA-Seq dataset GSE81089 was downloaded from GEO database (https://www.ncbi.nlm.nih.gov/geo/) with the inclusion criteria referring to the following microarray datasets. The publicly available TCPA-LUAD and TCPA-LUSC database (<https://www.tcpaportal.org/>) were used to determine the relationship between protein expression of some important genes and mRNA expression of Ki67.

Expression matrix and clinical data of 5 microarray datasets (GSE30219, GSE41271, GSE50081, GSE60486, GSE73403, GSE74777) which had more than 60 patients diagnosed as LUAD or LUSC with survival information were downloaded. Soft profiles were downloaded for probes annotation across different platforms and the probes IDs were matched to gene symbol. Median expression value was used as the expression of a gene matching to multiple probes.

10X single-cell RNA-Seq dataset GSE148071 containing 18 LUAD and 22 LUSC patients was downloaded from GEO database. R package "Seurat" version 4.3.0 removed the dead, double and broken cells according to criteria for filtration: (1) more than 200 as well as less than 5000 detected genes in the cells; (2) The proportion of mitochondrial genes in cells is less than 30%. After standardization, principal component dimensionality reduction clustering was performed based on the top 600 highly variable genes between cells. The R package "harmony"(33) was used to remove batch effects between samples. Finally, TSNE algorithm was used to divide and visualize the cell clusters based on the top 20 dimensions.

For public-access datasets, the endpoint of OS, DFS, RFS and PFS was defined as the time from surgery date to death from any cause, cancer-relates death, disease recurrence date and disease progression date. For the conflicting events between DFS, RFS, PFS and OS (patient died and had the same follow-up as OS), the endpoint would redefine using OS events as criteria.

**Gene set analysis**

To compare the biological functions of Ki67 between LUAD and LUSC, gene set enrichment analysis was carried out with Java desktop GSEA tool (version 4.0.1) against the KEGG, Hallmark and GO-BP collection in MsigDB (version 7.0). FDR adjusted p<0.05 and NES>1 simultaneously indicated significant annotations. We then downloaded 4 recognized proliferation associated pathways, enrolling “KEGG_CELL_CYCLE”, “REGULATION_OF_CELL_CYCLE”, “HALLMARK_E2F_TARGETS” and “BIOCARTA_CELLCYCLE_PATHWAY” from MsigDB to calculate the gene signature scores of each proliferative pathways across patients by gene set variation analysis (GSVA). Minimum size of the enrichment gene number was set as 10. Based on published data, we focused on a roster of 35 EMT-promoting genes (MYL9, TAGLN, ZEB2, MMP2, ACTA2, BGN, NNMT, SLIT3, GADD45B, AXL, TGM2, SPARC, ECM2, FAP, VIM, LOXL1, TIMP3, TGFB1, COL1A1, ZEB1, POSTN, MMP14, COL1A2, TGFB3, GEM, MXRA5, LOXL2, FN1, TIMP1, PCOLCE, GREM1, MMP7, GAS1, TGFB2, TWIST1) to curate into an EMT signature and to validate the result of GSEA.

**Differentiation trajectory**

Monocle2 R packages(34) was used to construct the differentiation trajectories of cancer cells in single-cell dataset. Differentially expressed genes (q<0.0001; expressed cells >200) of cancer cells in different proliferation group was screened by DifferentialGeneTest function. Based on the differentially expressed genes and the top 2 principal components of dimension, DDRTree algorithm inferred the differentiation trajectory and calculated the pseudo-time.

**Intercellular communication**

Based on the differentially overexpressed ligand and receptor genes between cell clusters in the single-cell dataset, as well as cell proportions, the interaction probabilities of co-activating ligand and co-inhibiting ligand were estimated using the Cellchat R package(35) to predict important intercellular communications, including secretory signals and cell-cell contact signals.

**Risk stratification and model construction**

Considering the putative factors influencing outcomes, we integrated Ki67 expression as continuous variable, EMT score and purity score into the survival *tree* analysis to identify the most variant subgroups. By constructing survival tree under 10-fold cross validation, the interactive elements which were hard to be detected in the traditional survival models could be effectively covered. The calibration curve was generated to evaluate the predictive accuracy of the nomogram model for OS at 1, 3 and 5 years. Nomogram and calibration curve were plotted by rms package. Time-dependent ROC curves(36) was applied to reflect the performance of the model predictive performance.

**References:**

1. Anami Y, Iijima T, Suzuki K, Yokota J, Minami Y, Kobayashi H*, et al.* Bronchioloalveolar carcinoma (lepidic growth) component is a more useful prognostic factor than lymph node metastasis. *Journal of thoracic oncology : official publication of the International Association for the Study of Lung Cancer* 2009;**4**(8):951-8.

2. Cui Y, Liu J, Yin H, Liu Y, Liu J. Fibulin-1 functions as a prognostic factor in lung adenocarcinoma. *Japanese journal of clinical oncology* 2015;**45**(9):854-9.

3. Del Gobbo A, Pellegrinelli A, Gaudioso G, Castellani M, Zito Marino F, Franco R*, et al.* Analysis of NSCLC tumour heterogeneity, proliferative and 18F-FDG PET indices reveals Ki67 prognostic role in adenocarcinomas. *Histopathology* 2016;**68**(5):746-51.

4. Demarchi L, Reis M, Palomino S, Farhat C, Takagaki T, Beyruti R*, et al.* Prognostic values of stromal proportion and PCNA, Ki-67, and p53 proteins in patients with resected adenocarcinoma of the lung. *Modern pathology : an official journal of the United States and Canadian Academy of Pathology, Inc* 2000;**13**(5):511-20.

5. Dong B, Sato M, Sakurada A, Sagawa M, Endo C, Wu S*, et al.* Computed tomographic images reflect the biologic behavior of small lung adenocarcinoma: they correlate with cell proliferation, microvascularization, cell adhesion, degradation of extracellular matrix, and K-ras mutation. *The Journal of thoracic and cardiovascular surgery* 2005;**130**(3):733-9.

6. Gasinska A, Kolodziejski L, Niemiec J, Dyczek S. Clinical significance of biological differences between cavitated and solid form of squamous cell lung cancer. *Lung cancer (Amsterdam, Netherlands)* 2005;**49**(2):171-9.

7. Glatzel-Plucinska N, Piotrowska A, Grzegrzolka J, Olbromski M, Rzechonek A, Dziegiel P*, et al.* SATB1 Level Correlates with Ki-67 Expression and Is a Positive Prognostic Factor in Non-small Cell Lung Carcinoma. *Anticancer research* 2018;**38**(2):723-36.

8. Haga Y, Hiroshima K, Iyoda A, Shibuya K, Shimamura F, Iizasa T*, et al.* Ki-67 expression and prognosis for smokers with resected stage I non-small cell lung cancer. *The Annals of thoracic surgery* 2003;**75**(6):1727-32; discussion 32-3.

9. Hashimoto K, Araki K, Osaki M, Nakamura H, Tomita K, Shimizu E*, et al.* MCM2 and Ki-67 expression in human lung adenocarcinoma: prognostic implications. *Pathobiology : journal of immunopathology, molecular and cellular biology* 2004;**71**(4):193-200.

10. Hayashi H, Ogawa N, Ishiwa N, Yazawa T, Inayama Y, Ito T*, et al.* High cyclin E and low p27/Kip1 expressions are potentially poor prognostic factors in lung adenocarcinoma patients. *Lung cancer (Amsterdam, Netherlands)* 2001;**34**(1):59-65.

11. Hokka D, Maniwa Y, Tane S, Nishio W, Yoshimura M, Okita Y*, et al.* Psf3 is a prognostic biomarker in lung adenocarcinoma. *Lung cancer (Amsterdam, Netherlands)* 2013;**79**(1):77-82.

12. Hommura F, Dosaka-Akita H, Mishina T, Nishi M, Kojima T, Hiroumi H*, et al.* Prognostic significance of p27KIP1 protein and ki-67 growth fraction in non-small cell lung cancers. *Clinical cancer research : an official journal of the American Association for Cancer Research* 2000;**6**(10):4073-81.

13. Ikeda J, Oda T, Inoue M, Uekita T, Sakai R, Okumura M*, et al.* Expression of CUB domain containing protein (CDCP1) is correlated with prognosis and survival of patients with adenocarcinoma of lung. *Cancer science* 2009;**100**(3):429-33.

14. Inoue M, Takakuwa T, Minami M, Shiono H, Utsumi T, Kadota Y*, et al.* Clinicopathologic factors influencing postoperative prognosis in patients with small-sized adenocarcinoma of the lung. *The Journal of thoracic and cardiovascular surgery* 2008;**135**(4):830-6.

15. Jethon A, Pula B, Olbromski M, Werynska B, Muszczynska-Bernhard B, Witkiewicz W*, et al.* Prognostic significance of SOX18 expression in non-small cell lung cancer. *International journal of oncology* 2015;**46**(1):123-32.

16. Kaira K, Oriuchi N, Imai H, Shimizu K, Yanagitani N, Sunaga N*, et al.* Prognostic significance of L-type amino acid transporter 1 (LAT1) and 4F2 heavy chain (CD98) expression in stage I pulmonary adenocarcinoma. *Lung cancer (Amsterdam, Netherlands)* 2009;**66**(1):120-6.

17. Kim C, Lee H, Park J, Choi J, Jang S, Park Y*, et al.* Prognostic role of p53 and Ki-67 immunohistochemical expression in patients with surgically resected lung adenocarcinoma: a retrospective study. *Journal of thoracic disease* 2015;**7**(5):822-33.

18. Maki Y, Soh J, Ichimura K, Shien K, Furukawa M, Muraoka T*, et al.* Impact of GLUT1 and Ki-67 expression on early‑stage lung adenocarcinoma diagnosed according to a new international multidisciplinary classification. *Oncology reports* 2013;**29**(1):133-40.

19. Mojtahedzadeh S, Hashimoto S, Nakashima Y, Koga T, Matsuo Y, Yoshino I*, et al.* Clinicopathologic relevance of apoptotic and proliferative factors in human lung adenocarcinoma: Fas expression correlates with the histologic subtype, but not with the degree of apoptosis. *Pathology, research and practice* 2002;**198**(10):645-54.

20. Oka S, Uramoto H, Shimokawa H, Iwanami T, Tanaka F. The expression of Ki-67, but not proliferating cell nuclear antigen, predicts poor disease free survival in patients with adenocarcinoma of the lung. *Anticancer research* 2011;**31**(12):4277-82.

21. Pawelczyk K, Piotrowska A, Ciesielska U, Jablonska K, Gletzel-Plucinska N, Grzegrzolka J*, et al.* Role of PD-L1 Expression in Non-Small Cell Lung Cancer and Their Prognostic Significance according to Clinicopathological Factors and Diagnostic Markers. *International journal of molecular sciences* 2019;**20**(4).

22. Pelosi G, Pasini F, Sonzogni A, Maffini F, Maisonneuve P, Iannucci A*, et al.* Prognostic implications of neuroendocrine differentiation and hormone production in patients with Stage I nonsmall cell lung carcinoma. *Cancer* 2003;**97**(10):2487-97.

23. Shimizu K, Maeda A, Yukawa T, Nojima Y, Saisho S, Okita R*, et al.* Difference in prognostic values of maximal standardized uptake value on fluorodeoxyglucose-positron emission tomography and cyclooxygenase-2 expression between lung adenocarcinoma and squamous cell carcinoma. *World journal of surgical oncology* 2014;**12**:343.

24. Shimoji M, Shimizu S, Sato K, Suda K, Kobayashi Y, Tomizawa K*, et al.* Clinical and pathologic features of lung cancer expressing programmed cell death ligand 1 (PD-L1). *Lung cancer (Amsterdam, Netherlands)* 2016;**98**:69-75.

25. Warth A, Cortis J, Soltermann A, Meister M, Budczies J, Stenzinger A*, et al.* Tumour cell proliferation (Ki-67) in non-small cell lung cancer: a critical reappraisal of its prognostic role. *British journal of cancer* 2014;**111**(6):1222-9.

26. Woo T, Okudela K, Mitsui H, Yazawa T, Ogawa N, Tajiri M*, et al.* Prognostic value of CD133 expression in stage I lung adenocarcinomas. *International journal of clinical and experimental pathology* 2010;**4**(1):32-42.

27. Wu Q, Qian Y, Zhao X, Wang S, Feng X, Chen X*, et al.* Expression and prognostic significance of centromere protein A in human lung adenocarcinoma. *Lung cancer (Amsterdam, Netherlands)* 2012;**77**(2):407-14.

28. Yazawa T, Kaira K, Shimizu K, Shimizu A, Mori K, Nagashima T*, et al.* Prognostic significance of β2-adrenergic receptor expression in non-small cell lung cancer. *American journal of translational research* 2016;**8**(11):5059-70.

29. Fujioka S, Shomori K, Nishihara K, Yamaga K, Nosaka K, Araki K*, et al.* Expression of minichromosome maintenance 7 (MCM7) in small lung adenocarcinomas (pT1): Prognostic implication. *Lung cancer (Amsterdam, Netherlands)* 2009;**65**(2):223-9.

30. Colaprico A, Silva TC, Olsen C, Garofano L, Cava C, Garolini D*, et al.* TCGAbiolinks: an R/Bioconductor package for integrative analysis of TCGA data. *Nucleic Acids Res* 2016;**44**(8):e71.

31. Liu J, Lichtenberg T, Hoadley KA, Poisson LM, Lazar AJ, Cherniack AD*, et al.* An Integrated TCGA Pan-Cancer Clinical Data Resource to Drive High-Quality Survival Outcome Analytics. *Cell* 2018;**173**(2):400-16 e11.

32. Zhang X, Wang J, Shao G, Wang Q, Qu X, Wang B*, et al.* Comprehensive genomic and immunological characterization of Chinese non-small cell lung cancer patients. *Nature communications* 2019;**10**(1):1772.

33. Korsunsky I, Millard N, Fan J, Slowikowski K, Zhang F, Wei K*, et al.* Fast, sensitive and accurate integration of single-cell data with Harmony. *Nature Methods* 2019;**16**(12):1289-96.

34. Trapnell C, Cacchiarelli D, Grimsby J, Pokharel P, Li S, Morse M*, et al.* The dynamics and regulators of cell fate decisions are revealed by pseudotemporal ordering of single cells. *Nature Biotechnology* 2014;**32**(4):381-6.

35. Jin S, Guerrero-Juarez CF, Zhang L, Chang I, Ramos R, Kuan C-H*, et al.* Inference and analysis of cell-cell communication using CellChat. *Nature Communications* 2021;**12**(1):1088.

36. P B, JF D, H J-G. Estimating and comparing time-dependent areas under receiver operating characteristic curves for censored event times with competing risks. *Statistics in medicine* 2013;**32**(30):5381-97.

**Supplementary tables**

Table S1. Clinicopathological parameters of patients from our cohort.

| **Characteristics** | **All (n=1479)** | **LUAD (n=1116)** | **LUSC (n=363)** |
| --- | --- | --- | --- |
| Gender, n (%) |  |  |  |
| Male | 918 (62.07%) | 593 (53.14%) | 325 (89.53%) |
| Female | 561 (37.93%) | 523 (46.86%) | 38 (10.47%) |
| Age, years |  |  |  |
| Mean | 58.59 | 58.23 | 59.7 |
| Range | 26-85 | 26-85 | 31-76 |
| <65 years old | 1096 (73.07%) | 834 (74.73%) | 256 (70.52%) |
| ≥65 years old | 389 (25.93%) | 269 (24.10%) | 105 (28.93%) |
| No data | 15 (1.00%) | 13 (1.16%) | 2 (0.55%) |
| Smoking History, n (%) |  |  |  |
| Yes | 700 (47.33%) | 416 (37.28%) | 284 (78.24%) |
| No | 774 (52.33%) | 697 (62.46%) | 77 (21.21%) |
| No data | 5 (0.34%) | 3 (0.27%) | 2 (0.55%) |
| Family History, n (%) |  |  |  |
| Yes | 424 (28.67%) | 316 (28.32%) | 108 (29.75%) |
| No | 1053 (71.20%) | 798 (71.51%) | 255 (70.25%) |
| No data | 2 (0.14%) | 2 (0.18%) | - |
| Malignancy Grade, n (%) |  |  |  |
| G1-G2 | 294 (19.88%) | 186 (16.67%) | 108 (29.75%) |
| G3-G4 | 211 (14.27%) | 124 (11.11%) | 87 (23.97%) |
| No data | 974 (65.86%) | 806 (72.22%) | 168 (46.28%) |
| Tumor Size, n (%) |  |  |  |
| T1 | 242 (16.36%) | 206 (18.46%) | 36 (9.92%) |
| T2 | 555 (37.53%) | 402 (36.02%) | 153 (42.15%) |
| T3 | 233 (15.75%) | 150 (13.44%) | 83 (22.87%) |
| T4 | 332 (22.45%) | 272 (24.37%) | 60 (16.53%) |
| Tis | 1 (0.07%) | 1 (0.09%) | 0 (0.00%) |
| No data | 116 (7.84%) | 85 (7.62%) | 31 (8.54%) |
| Lymph Nodes, n (%) |  |  |  |
| N0 | 306 (20.69%) | 232 (20.79%) | 74 (20.39%) |
| N1 | 244 (16.50%) | 154 (13.80%) | 90 (24.79%) |
| N2 | 442 (29.89%) | 340 (30.47%) | 102 (28.10%) |
| N3 | 317 (21.43%) | 268 (24.01%) | 49 (13.50%) |
| No data | 170 (11.49%) | 122 (10.93%) | 48 (13.22%) |
| Stage, n (%) |  |  |  |
| I-IIIA | 642 (43.41%) | 417 (37.37%) | 225 (61.98%) |
| IIIB-IV  No data | 826 (55.85%)  11 (0.74%) | 692 (62.00%)  7 (0.63%) | 134 (36.91%)  4 (1.10%) |

“No data” and “-” represent the information is not available; also applied in table S2, S3 and S4.

Table S2. Univariate and multivariate Cox-regression of LUAD patients from our cohort with OS as endpoint.

| **Clinicopathological parameters** | **Univariate analysis** | | | | **Multivariate analysis** | | |
| --- | --- | --- | --- | --- | --- | --- | --- |
|  | **HR** | **95%CI** | | ***P-value*** | **HR** | **95%CI** | ***P-value*** |
| Age |  |  |  | |  |  |  |
| ≥65 *vs* <65 | 0.408 | 0.152-1.091 | 0.074 | | 0.959 | 0.792-1.163 | 0.673 |
| No data *vs* <65 | 0.551 | 0.305-0.996 | **0.048** | | 0.547 | 0.204-1.468 | 0.231 |
| Gender |  |  |  | |  |  |  |
| Female *vs* Male | 0.755 | 0.651-0.874 | **<0.001** | | 0.959 | 0.792-1.163 | 0.673 |
| Smoking History |  |  |  | |  |  |  |
| Have *vs* No | 1.432 | 1.236-1.659 | **<0.001** | | 1.411 | 1.165-1.710 | **<0.001** |
| Family History |  |  |  | |  |  |  |
| Have *vs* No | 0.979 | 0.829-1.155 | 0.8 | | - | - | - |
| Malignancy Grade |  |  |  | |  |  |  |
| G3-G4 *vs* G1-G2 | 1.789 | 1.426-2.245 | **<0.001** | | 1.201 | 0.944-1.527 | 0.135 |
| No data *vs* G1-G2 | 0.565 | 0.435-0.735 | **<0.001** | | 0.748 | 0.571-0.979 | **0.034** |
| TNM Stage |  |  |  | |  |  |  |
| IIIB-IV *vs* I-III | 2.685 | 2.251-3.203 | **<0.001** | | 2.597 | 2.158-3.126 | **<0.001** |
| Ki67 expression |  |  |  | |  |  |  |
| ≥25% *vs* <25% | 1.536 | 1.326-1.780 | **<0.001** | | 1.384 | 1.191-1.609 | **<0.001** |

Table S3. Univariate and multivariate Cox-regression of LUSC patients from our cohort with OS as endpoint.

| **Clinicopathological parameters** | **Univariate analysis** | | | | **Multivariate analysis** | | |
| --- | --- | --- | --- | --- | --- | --- | --- |
|  | **HR** | **95%CI** | | ***P-value*** | **HR** | **95%CI** | ***P-value*** |
| Age |  |  |  | |  |  |  |
| ≥65 *vs* <65 | 0.796 | 0.198-3.208 | 0.749 | | - | - | - |
| No data *vs* <65 | 0.678 | 0.293-1.567 | 0.363 | | - | - | - |
| Gender |  |  |  | |  |  |  |
| Female *vs* Male | 1.171 | 0.818-1.674 | 0.388 | | - | - | - |
| Smoking History |  |  |  | |  |  |  |
| Have *vs* No | 0.759 | 0.186-3.094 | 0.701 | | - | - | - |
| No data *vs* No | 1.197 | 0.524-2.733 | 0.670 | | - | - | - |
| Family History |  |  |  | |  |  |  |
| Have *vs* No | 0.971 | 0.755-1.249 | 0.819 | | - | - | - |
| Malignancy Grade |  |  |  | |  |  |  |
| G3-G4 *vs* G1-G2 | 2.399 | 1.788-3.219 | **<0.001** | | 1.846 | 1.355-2.516 | **<0.001** |
| No data *vs* G1-G2 | 1.218 | 0.876-1.693 | 0.242 | | 1.167 | 0.839-1.624 | 0.359 |
| TNM Stage |  |  |  | |  |  |  |
| IIIB-IV *vs* I-IIIA | 2.679 | 2.109-3.404 | **<0.001** | | 2.228 | 1.732-2.868 | **<0.001** |
| Ki67 expression |  |  |  | |  |  |  |
| ≥25% *vs* <25% | 0.711 | 0.533-0.948 | **0.020** | | 0.760 | 0.570-1.015 | **0.063** |

Table S4. Log-rank test and landmark analysis of LUSC patients with DFS, RFS and PFS as endpoint.

| **LUSC**  **datasets** | **DFS** | | | |  | **RFS** | | | |  | **PFS** | |
| --- | --- | --- | --- | --- | --- | --- | --- | --- | --- | --- | --- | --- |
|  | **GSE30219**  **(x=72)** | | **TCGA**  **(x=60)** | |  | **GSE74777**  **(x=48)** | | **GSE41271**  **(x=48)** | |  | **TCGA**  **(x=60)** | |
|  | **HR** | ***P-value*** | **HR** | ***P-value*** |  | **HR** | ***P-value*** | **HR** | ***P-value*** |  | **HR** | ***P-value*** |
| All | 0.676 | 0.192 | 0.908 | 0.617 |  | 1.500 | 0.262 | 1.156 | 0.641 |  | 0.890 | 0.358 |
| ≤ x (months) | 0.631 | 0.163 | 0.821 | 0.327 |  | 1.820 | 0.161 | 0.919 | 0.783 |  | 0.854 | 0.238 |
| > x (months) | 0.263 | 0.051 | 0.350 | 0.095 |  | 0.683 | 0.598 | - | - |  | 0.476 | 0.048* |

Table S5. Top 5 enriched pathways of Ki67 (NES>1 & FDR q-value<0.25).

| **Gene set** | **Correlation** | **Histology** | **Top 5 enriched pathways** |
| --- | --- | --- | --- |
| Hallmark | Positive | LUAD | HALLMARK_G2M_CHECKPOINT  HALLMARK_MITOTIC_SPINDLE  HALLMARK_E2F_TARGETS  HALLMARK_MTORC1_SIGNALING  HALLMARK_MYC_TARGETS_V1 |
|  |  | LUSC | HALLMARK_MITOTIC_SPINDLE  HALLMARK_G2M_CHECKPOINT  HALLMARK_E2F_TARGETS  HALLMARK_MYC_TARGETS_V2  HALLMARK_SPERMATOGENESIS |
|  | Negative | LUAD | - |
|  |  | LUSC | HALLMARK_COAGULATION |
| KEGG | Positive | LUAD | KEGG_CELL_CYCLE  KEGG_OOCYTE_MEIOSIS  KEGG_PROGESTERONE_MEDIATED_OOCYTE_MATURATION  KEGG_DNA_REPLICATION  KEGG_ONE_CARBON_POOL_BY_FOLATE |
|  |  | LUSC | KEGG_CELL_CYCLE  KEGG_PROGESTERONE_MEDIATED_OOCYTE_MATURATION  KEGG_LYSINE_DEGRADATION  KEGG_HOMOLOGOUS_RECOMBINATION  KEGG_PROSTATE_CANCER |
|  | Negative | LUAD | KEGG_ARACHIDONIC_ACID_METABOLISM  KEGG_PRIMARY_BILE_ACID_BIOSYNTHESIS  KEGG_LINOLEIC_ACID_METABOLISM  KEGG_ASTHMA  KEGG_DRUG_METABOLISM_CYTOCHROME_P450 |
|  |  | LUSC | KEGG_COMPLEMENT_AND_COAGULATION_CASCADES  KEGG_PRIMARY_BILE_ACID_BIOSYNTHESIS  KEGG_GLYCOSPHINGOLIPID_BIOSYNTHESIS_GANGLIO_SERIES  KEGG_LYSOSOME  KEGG_OTHER_GLYCAN_DEGRADATION |
| GO-BP | Positive | LUAD | GO_MITOTIC_NUCLEAR_DIVISION  GO_REGULATION_OF_DNA_METABOLIC_PROCESS  GO_CHROMOSOME_SEGREGATION  GO_MITOTIC_SPINDLE_ORGANIZATION  GO_POSITIVE_REGULATION_OF_DNA_METABOLIC_PROCESS |
|  |  | LUSC | GO_MITOTIC_NUCLEAR_DIVISION  GO_CHROMOSOME_SEGREGATION  GO_SISTER_CHROMATID_SEGREGATION  GO_SPINDLE_ORGANIZATION  GO_NUCLEAR_CHROMOSOME_SEGREGATION |
|  | Negative | LUAD | GO_WATER_TRANSPORT  GO_FLUID_TRANSPORT  GO_RENAL_ABSORPTION  GO_COMPLEMENT_ACTIVATION |
|  |  | LUSC | GO_NEGATIVE_REGULATION_OF_COAGULATION  GO_COMPLEMENT_ACTIVATION  GO_REGULATION_OF_HUMORAL_IMMUNE_RESPONSE  GO_PHAGOSOME_ACIDIFICATION  GO_AZOLE_TRANSPORT |

**Supplementary Figures**

Figure S1. Forest plots of published studies on evaluating prognostic value of Ki67 in LUAD and LUSC.

(A-B) Meta-analysis (A) and cumulative meta-analysis (B) in LUAD with OS as endpoint.

(C-D) Meta-analysis (C) and cumulative meta-analysis (D) in LUAD with DFS as endpoint.

(E-F) Meta-analysis (E) and cumulative meta-analysis (F) in LUSC with OS as endpoint.


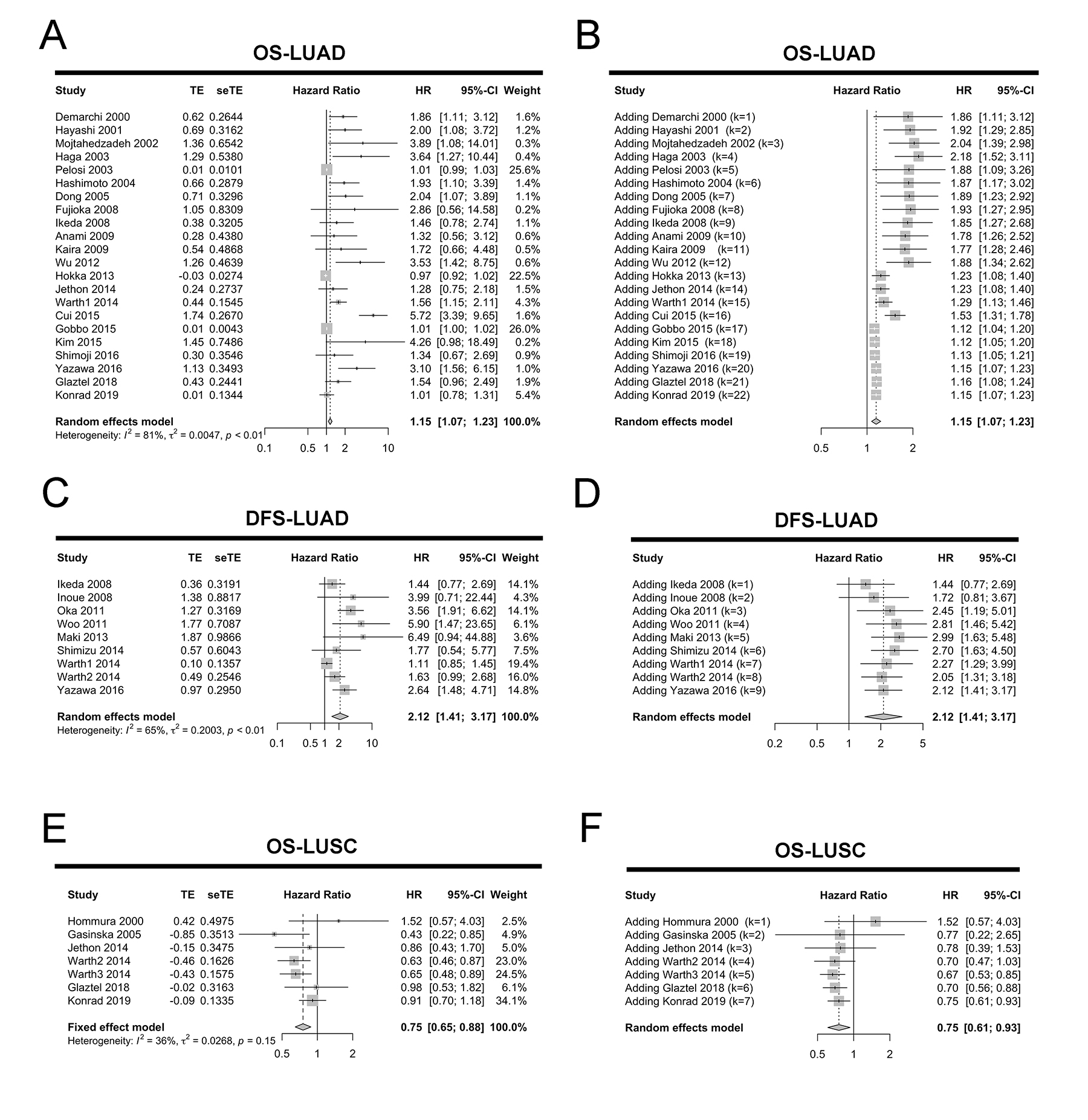


Figure S2. Sensitivity analysis and bias correction of meta-analysis.

(A-C) Sensitivity analysis based on year of publication, (D-F) detection of publication bias and (G-I) bias correction through trim-fill method.


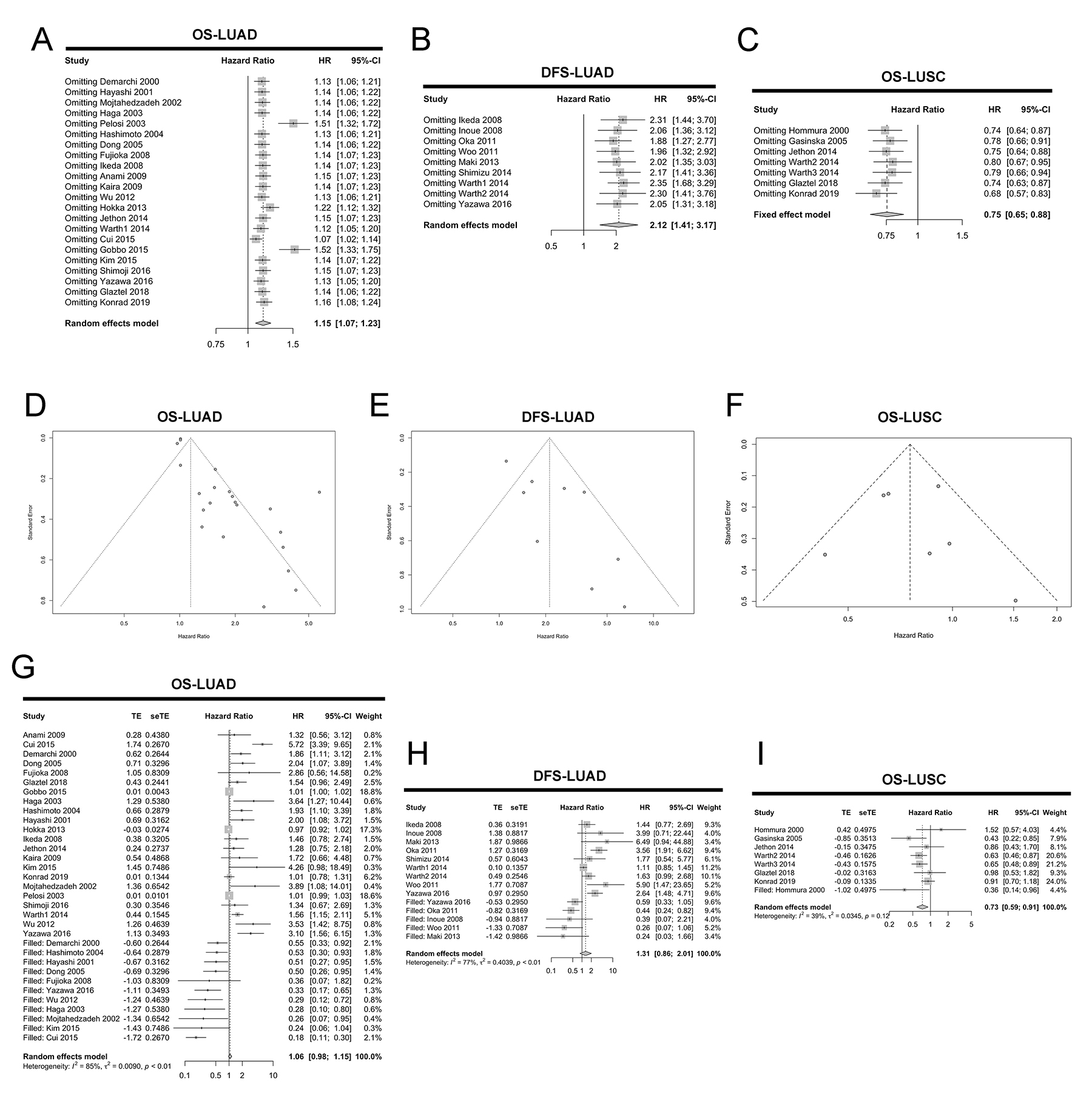


Figure S3. Kaplan-Meier analysis and landmark analysis of overall-survival.

(A-C) Prognostic analysis of Ki67 mRNA expression in LUAD patients from GSE41271, GSE81089 and TCGA cohort.

(D-K) Prognostic analysis of Ki67 mRNA expression in LUSC patients from (D-E) TCGA, (F-G) GSE30219, (H-I) GSE74777, (J-K) GSE41271 and (L-M) GSE81089. The time-point for landmark analysis was 66-month, 72-month, 60-month, 60-month and 42-month respectively.


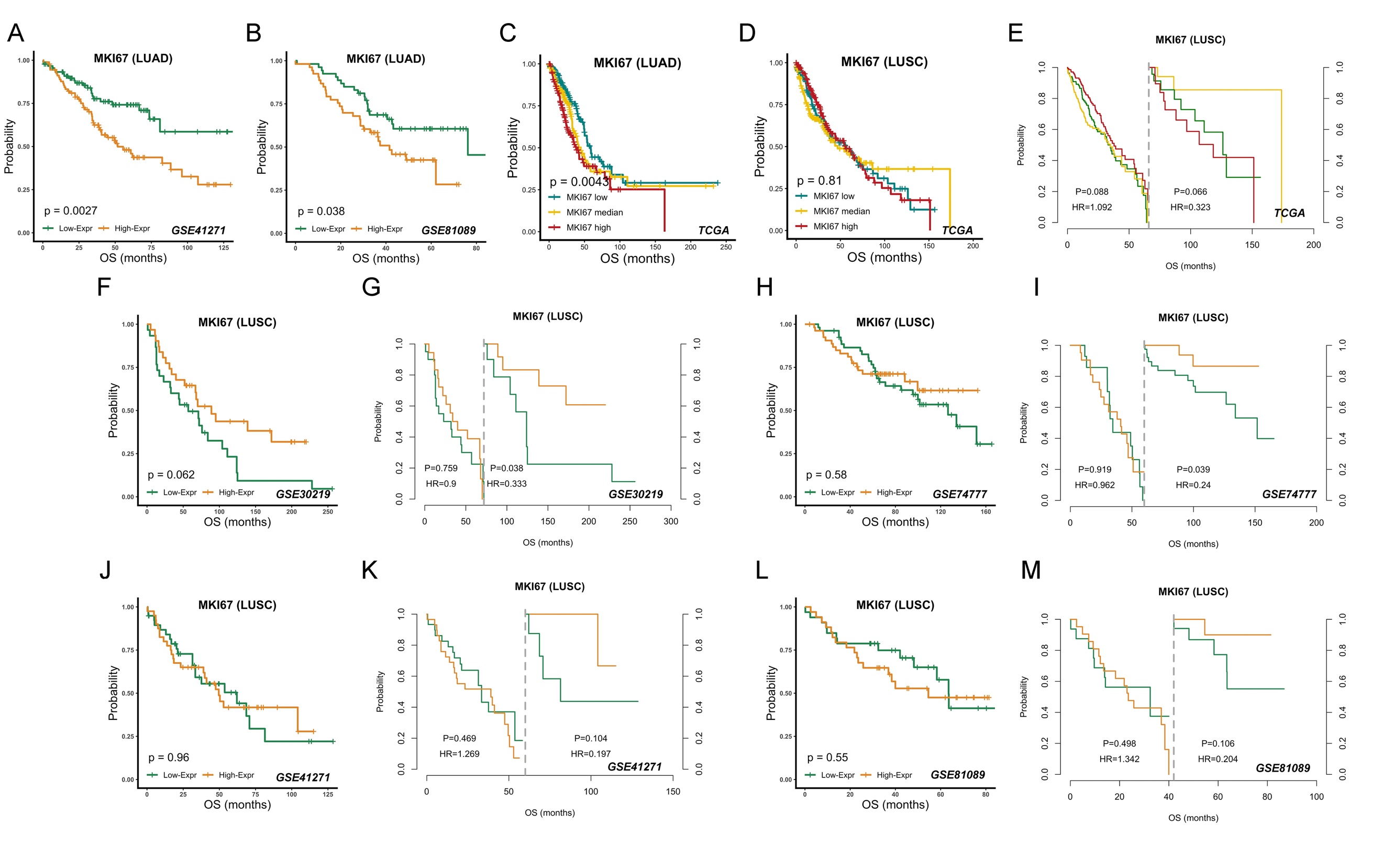


Figure S4. Univariate cox-Regression analysis of Ki67 under different cutoff value in our cohort and comparison of baseline characteristics among the LUSC-related datasets.

(A-B) Beta-value (the Natural logarithm of HR) and -log10 P-value showed in our cohort. Beta-value > 0 implied poor prognosis in group of high Ki67 expression and -log10 *P*-value > 1.301 indicated significant result.

(C-D) Baseline characteristic comparison of (C) microarray, RNA-Seq datasets and (D) IHC datasets including meta-analysis and our cohort. (“0%” is representative of unavailable information in the corresponding datasets.)


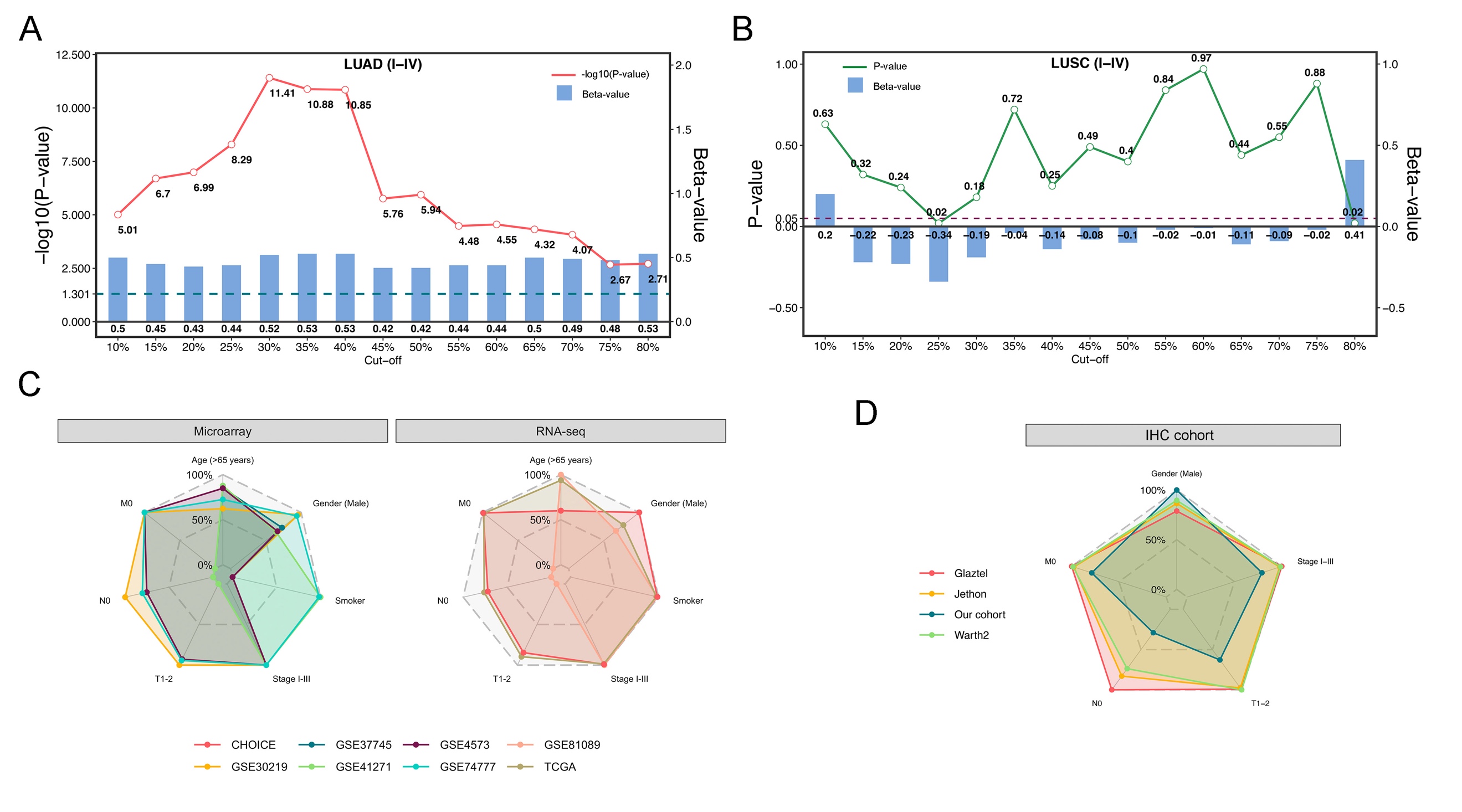


Figure S5. Relationship of Ki67 and proliferative activity in TCGA cohort.

(A) Pearson correlation test between Ki67 expression and scores of 4 proliferative gene-sets.

(B) Overlap of proliferative gene-sets and Ki67 correlated genes (r≥0.5, *P*<0.001).

(C) Dysregulated degree of HALLMARK_OF_CELL_CYCLE pathway reflected by the expression of Ki67 and the correlated genes (r≥0.5, *P*<0.001).

(D) Comparison of HALLMARK_OF_CELL_CYCLE pathway dysregulated scores between LUAD and LUSC according to cumulative distribution.

(E-F) The same analysis performed on HALLMARK_E2F_TARGETS pathway.


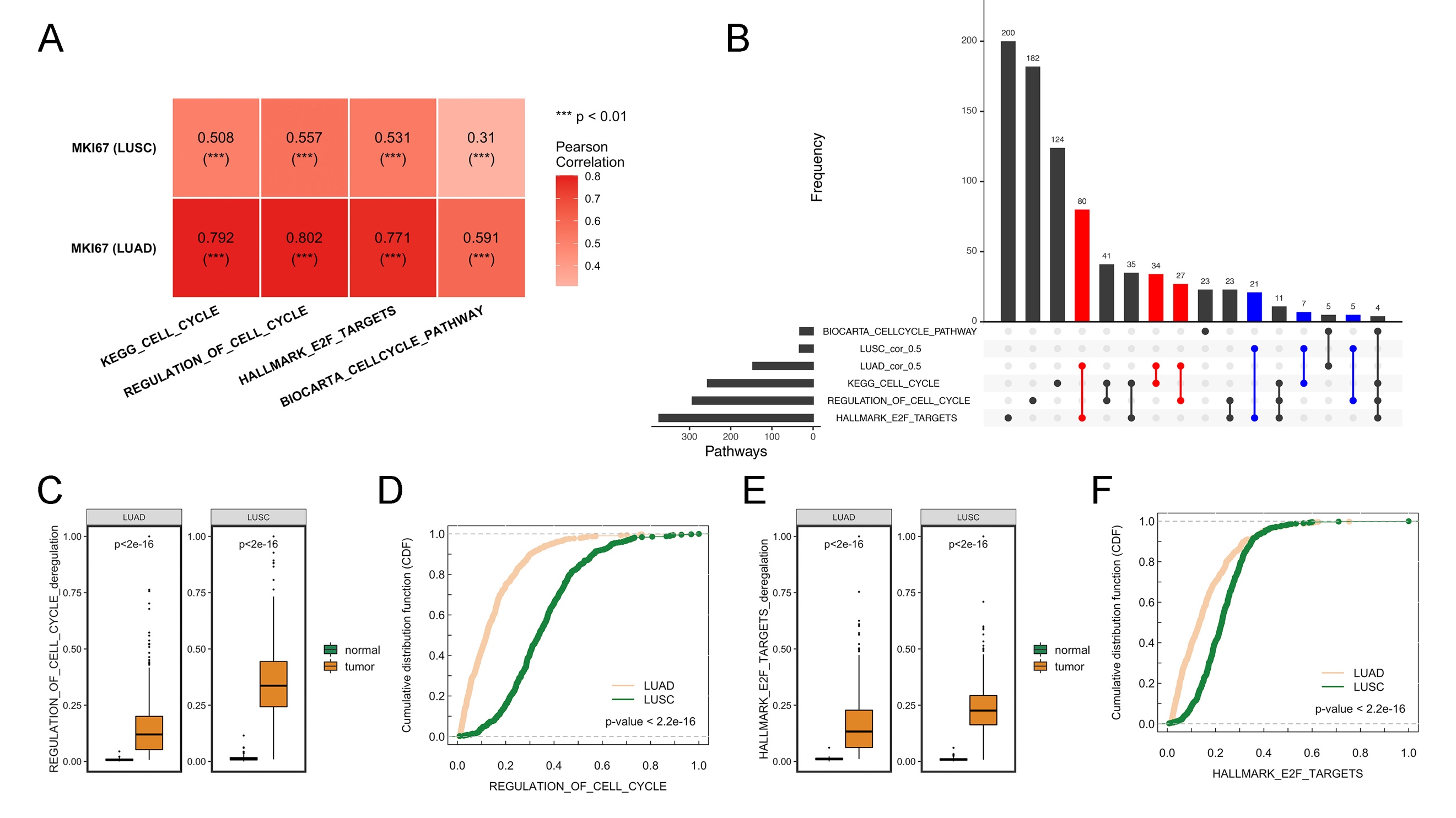


Figure S6. Prognostic evaluation of proliferative subgroups in TCGA-LUAD cohort.


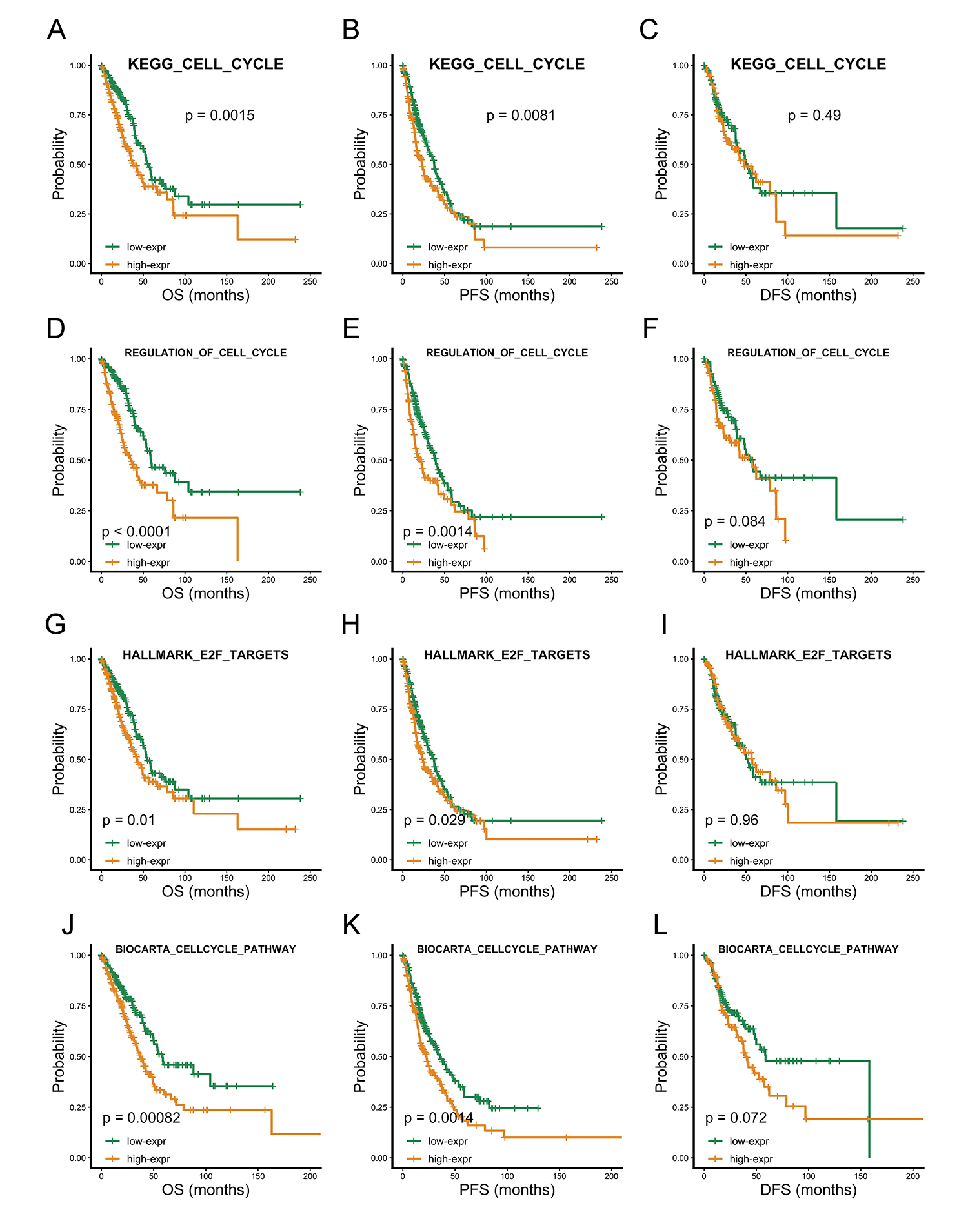


Figure S7. Prognostic evaluation of proliferative subgroups and EMT aberration in TCGA-LUSC.

(A-I) Prognostic comparison of subgroups classified by proliferative score based on different proliferative pathways.

(J) Prognostic comparison of patients stratified by EMT signature score with OS as endpoint.


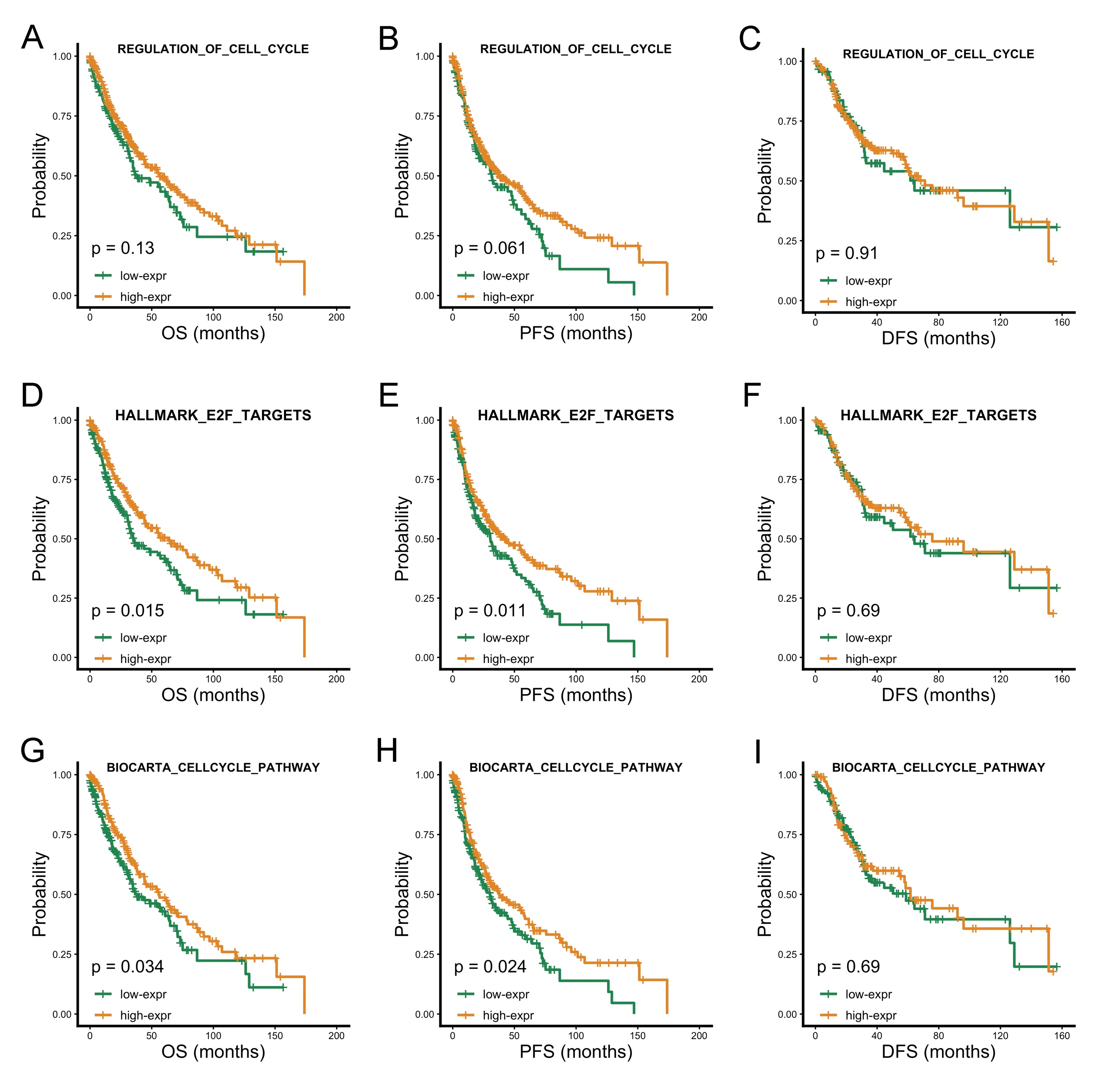


Figure S8. Annotation of cell clusters in single-cell dataset and analysis of LUAD tumor microenviroment.

(A-B) Expression of marker genes in cell clusters of (A) LUSC and (B) LUAD.

(C) Differentiation trajectory of LUAD cancer cells grouped by proliferative capacity in the scRNA-Seq dataset.

(D) Cell proportion of LUAD tumors of different trajectories in the scRNA-Seq dataset (branch one: n=2, branch two: n=5, branch three: n=1, mix branch: n=10).

(E) Wilcoxon test of immune cells in different proliferating groups in TCGA-LUAD, including the proportion of M2-macrophage in all macrophages and neutrophils, as well as stromal cells including CAFs and endothelial cells.


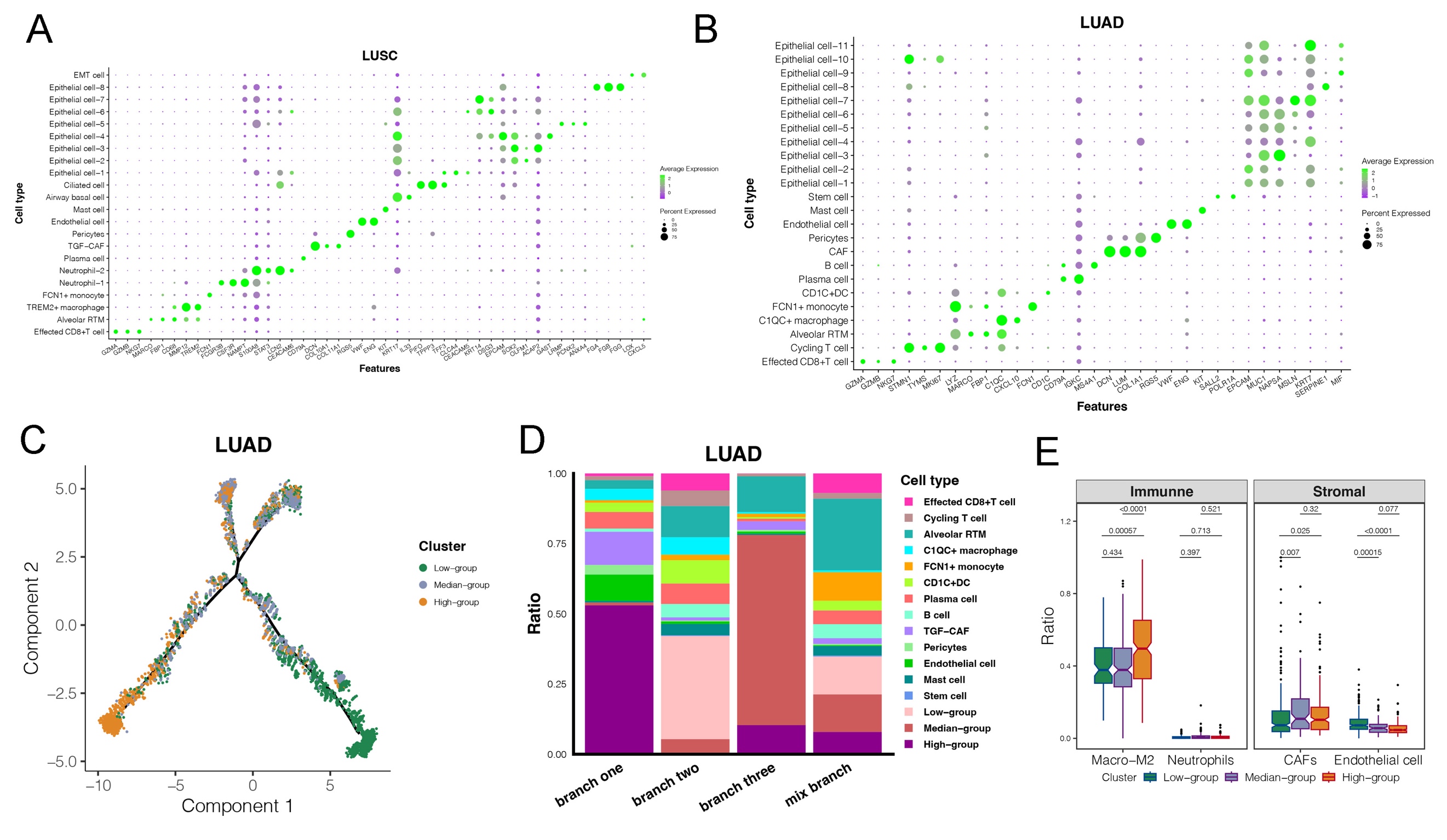


Figure S9. Relationship of proliferative activity, EMT potential and immune status in GSE30219.

(A-D) Prognostic comparison of subgroups classified by KEGG_CELL_CYCLE proliferative score in (A-B) LUSC patients and (C-D) LUAD patients. Analysis was performed with OS and DFS as endpoint.

(E-F) Pearson correlation test between the expression of Ki67 and 35 EMT-promoting genes in (E) LUSC and (F) LUAD (**P*<0.01).

(G-J) Degree of tumor purity, infiltration of CAFs and endothelial cells in (G-H) LUSC and in (I-J) LUAD.

(K-L) Enrichment score of immune cells in (K) LUSC and (L) LUAD (*****P*<1e-04, ****P*<0.001, ***P*<0.01, **P*<0.05, ns *P*>0.05).

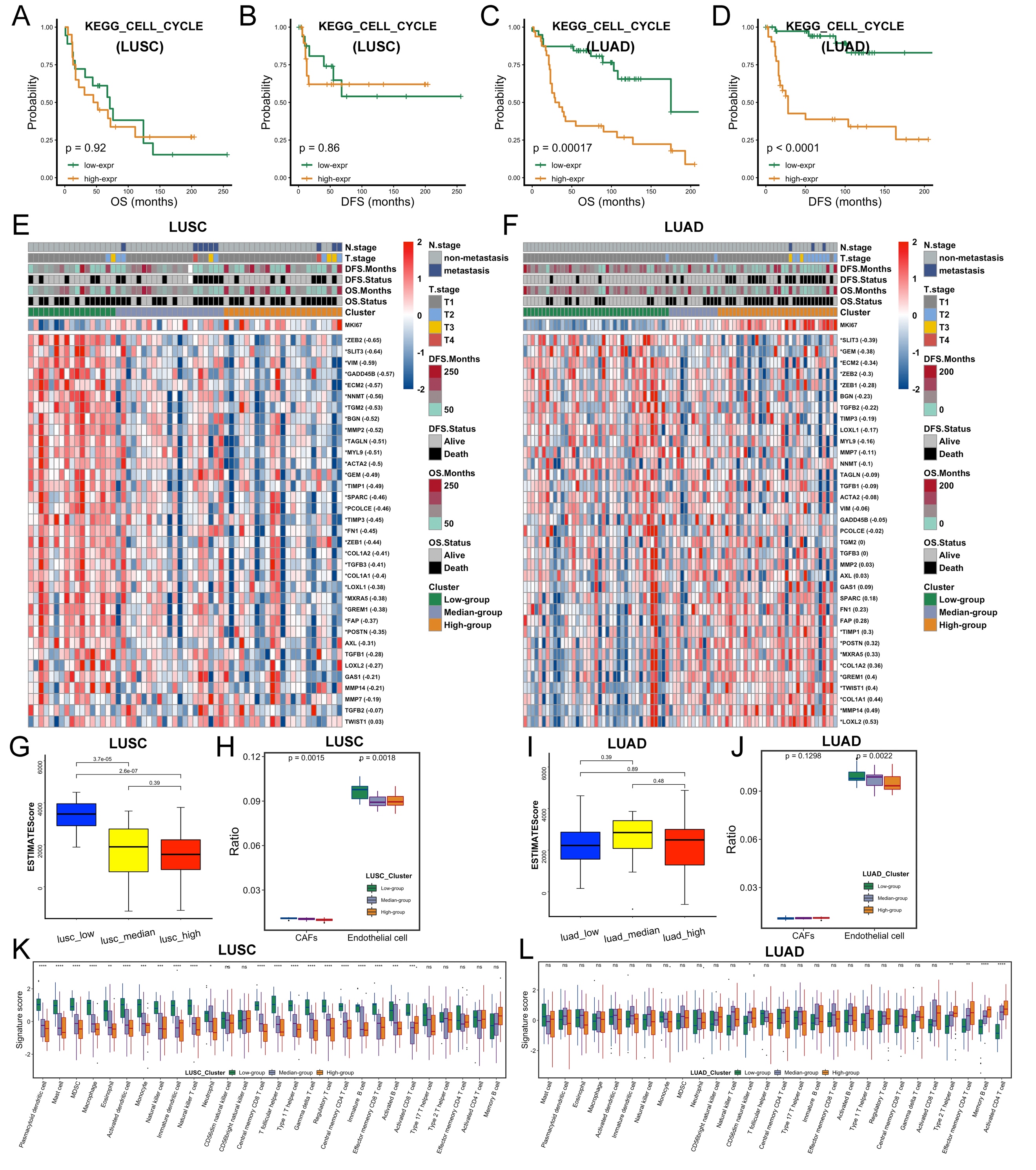


Figure S10. Relationship of proliferative activity, EMT potential and immune status in GSE81089.


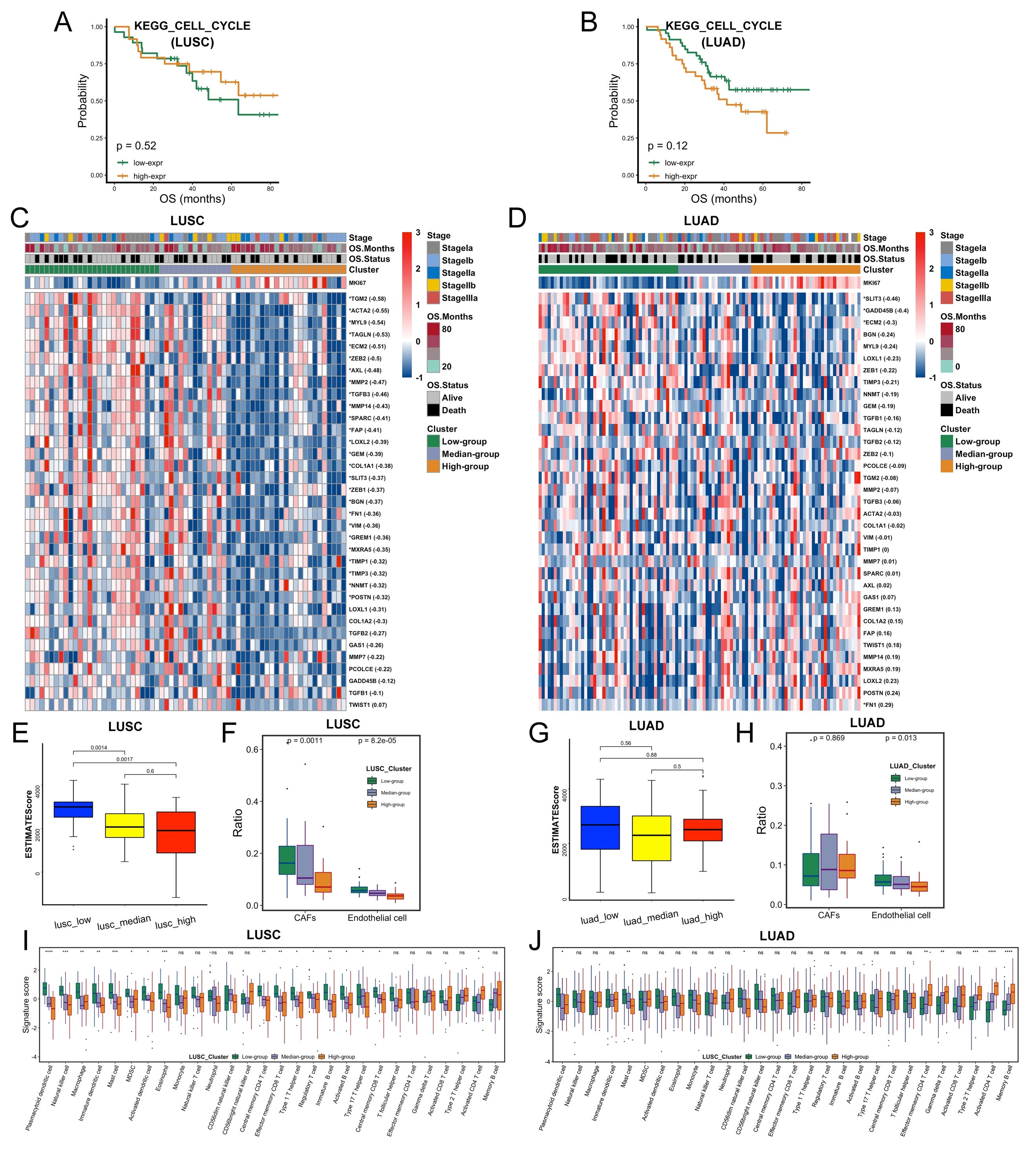


Figure S11. Relationship of proliferative activity, EMT potential and immune status in CHOICE cohort.


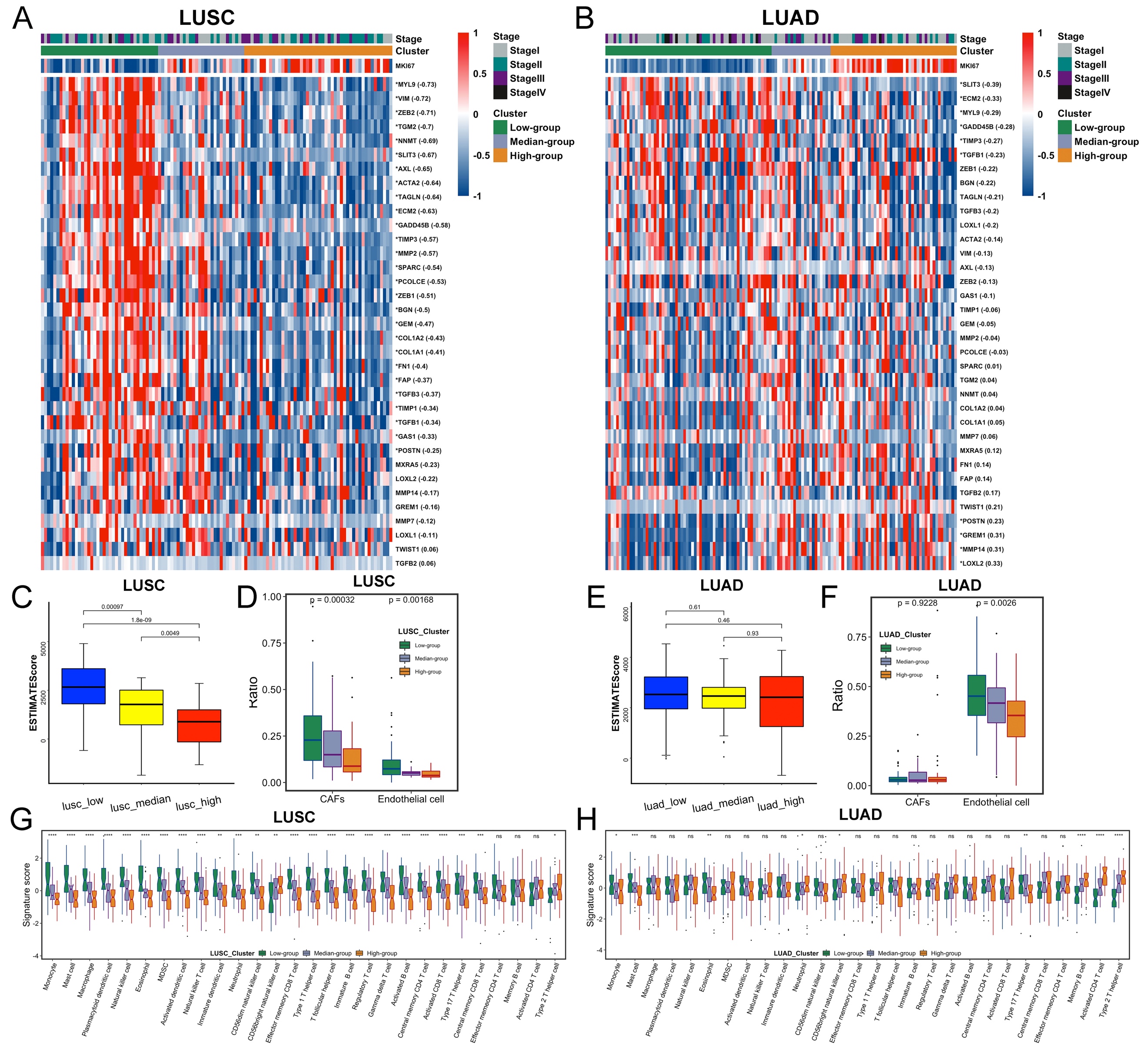


Figure S12. Survival tree analysis in LUSC with OS as endpoint.

Risk stratification and prognostic comparison of patients discriminated by survival tree analysis with OS occurring before and after 5 years in (A-B) GSE30219, (C-D) GSE74777, (E-F) GSE60486 and (G-H) GSE41271. The stratified time-point in (I-J) GSE73403 and (K-L) GSE81089 was 2.5 years.


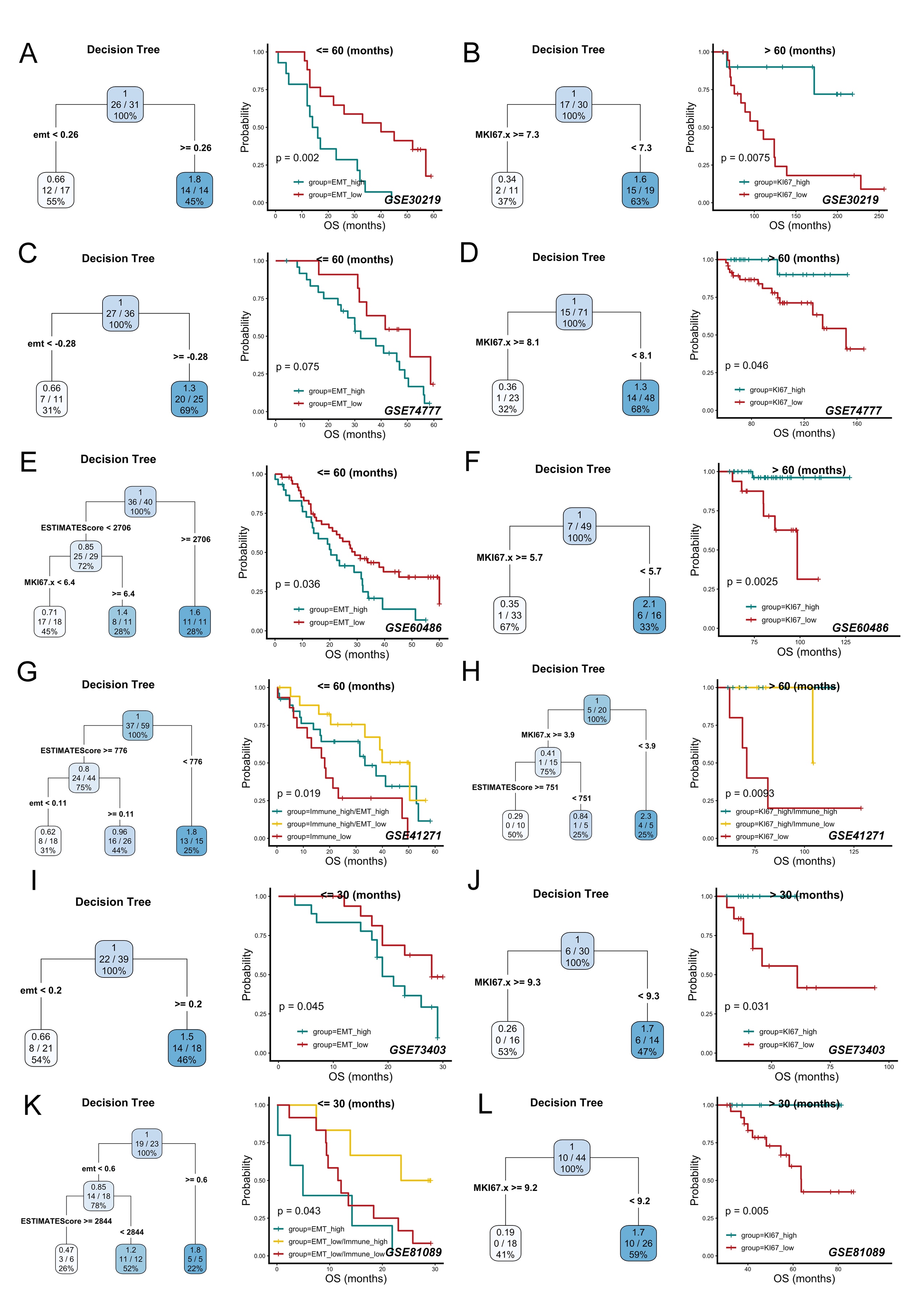


Figure S13. Survival tree analysis in LUSC with DFS and PFS as endpoint.

Risk stratification and prognostic comparison of patients discriminated by survival tree analysis with DFS occurring before and after 5 years in (A-D) GSE30219, (E-H) TCGA and analysis with PFS as endpoint in (I-L) TCGA.


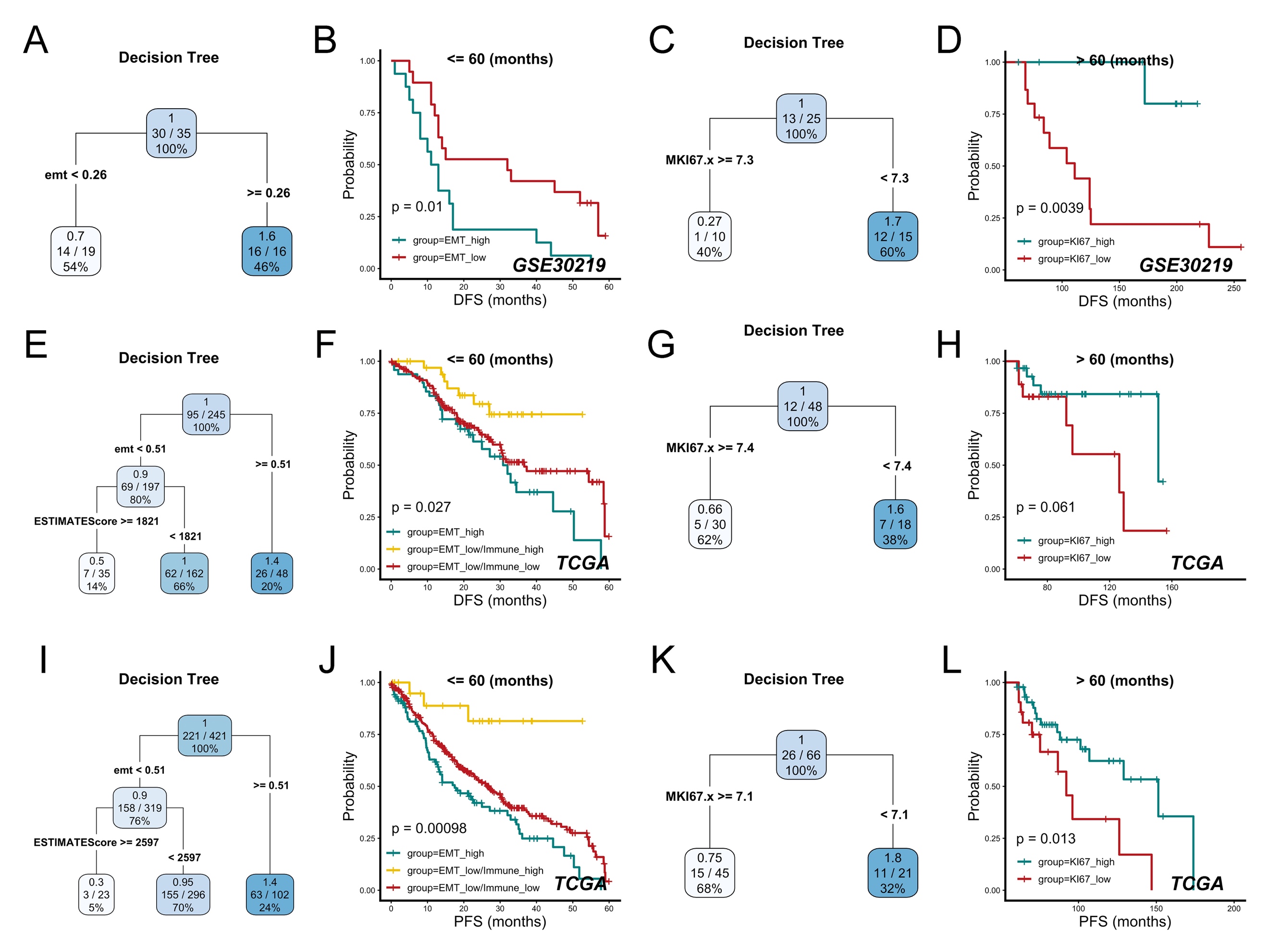

Supplement: Supplementary file 1 — Appendix S1. [file JCMM-28-e18521-s001.docx]
